# Gene signatures in patients with early breast cancer and relapse despite pathologic complete response

**DOI:** 10.1038/s41523-022-00403-3

**Published:** 2022-03-29

**Authors:** Simona Bruzas, Oleg Gluz, Nadia Harbeck, Peter Schmid, Javier Cortés, Jens Blohmer, Christine Seiberling, Ouafaa Chiari, Hakima Harrach, Beyhan Ataseven, Satyendra Shenoy, Mark H. Dyson, Eugen Traut, Ingo Theuerkauf, Daniel Gebauer, Sherko Kuemmel, Mattea Reinisch

**Affiliations:** 1 Interdisciplinary Breast Unit, Kliniken Essen-Mitte, Essen, Germany; 2 West German Study Group, Moenchengladbach, Germany; 3 Breast Center Niederrhein, Evangelical Hospital Bethesda, Moenchengladbach, Germany; 4 Breast Center, Dept. OB&GYN and CCCLMU, University of Munich (LMU), Munich, Germany; 5 Barts Cancer Institute, Queen Mary University London, London, United Kingdom; 6 International Breast Cancer Centre (IBCC), Quiron Group, Barcelona, Spain; 7 Vall d’Hebron Institute of Oncology (VHIO), Barcelona, Spain; 8 Medica Scientia Innovation Research (MEDSIR), Barcelona, Spain; 9 Medica Scientia Innovation Research (MEDSIR), Ridgewood, NJ USA; 10 Klinik für Gynäkologie mit Brustzentrum, Charité-Universitätsmedizin Berlin, Berlin, Germany; 11 Clinic for Gynecology and Gynecological Oncology, Kliniken Essen-Mitte, Essen, Germany; 12 Institut für Pathologie Viersen, Viersen, Germany

**Keywords:** Prognostic markers, Tumour biomarkers

## Abstract

A substantial minority of early breast cancer (EBC) patients relapse despite their tumors achieving pathologic complete response (pCR) after neoadjuvant therapy. We compared gene expression (BC360; nCounter^®^ platform; NanoString) between primary tumors of patients with post-pCR relapse (*N* = 14) with: (i) matched recurrent tumors from same patient (intraindividual analysis); and (ii) primary tumors from matched controls with pCR and no relapse (*N* = 41; interindividual analysis). Intraindividual analysis showed lower estrogen receptor signaling signature expression in recurrent tumors versus primaries (logFC = −0.595; *P* = 0.022). Recurrent tumors in patients with distant metastases also exhibited reduced expression of immune-related expression parameters. In interindividual analyses, primary tumor major histocompatibility complex class II expression was lower versus controls in patients with any relapse (logFC = −0.819; *P* = 0.030) or distant relapse (logFC = −1.151; *P* = 0.013). Primaries with later distant relapse also had greater homologous recombination deficiency than controls (logFC = 0.649; *P* = 0.026). Although no associations remained statistically significant following adjustment for false discovery rate, our results show that transcriptomic analyses have potential for prognostic value and may help in selecting optimal treatment regimens for EBC at risk of relapse and warrant further investigation.

## Introduction

The management paradigm for early breast cancer (EBC) is shifting away from adjuvant treatment towards neoadjuvant strategies as a standard of care for patients with more aggressive subtypes^1^. Originally used for downstaging locally advanced tumors, neoadjuvant systemic therapy (NST) has established benefits in improving surgical outcomes by increasing operability, facilitating breast-conserving surgery, and reducing the extent of lymph node resection^2,3^. Furthermore, NST enables in vivo chemosensitivity testing to inform about prognostication and guide subsequent treatment decisions based on individual response^4^. The overall prognosis in patients who attain pathologic complete response (pCR) is exceptional^5^ and the highest pCR rates are observed in patients with triple-negative breast cancer (TNBC) and human epidermal growth factor receptor-2 (HER2)-positive subtypes; in whom the association between pCR and improved outcomes is also strongest^5,6^. Meanwhile, for patients not achieving pCR, a new model of post-neoadjuvant treatment escalation is emerging^7^, with recent trials demonstrating benefit in this setting for adjuvant capecitabine for TNBC and trastuzumab emtansine for HER2-positive disease^8,9^.

Despite the positive prognostic impact of invasive disease eradication, a significant minority of patients with pCR following NST ultimately relapse^10^. In a large analysis conducted by the German Breast Group on a database which included 2188 patients with pCR from five neoadjuvant trials, the rate of disease-free survival (DFS) at 5 years was reported to be 87%^11^. Similarly, a recent meta-analysis reported a 5-year event-free survival (EFS) of 88% among 5748 pCR-attaining patients from clinical trials or cohort studies^5^. In that analysis, 5-year EFS in patients with pCR was lower in TNBC (90%) or HER2-positive subtypes (86%) than in hormone receptor (HR)-positive disease (97%). Relapse risk in patients with pCR has been associated with several clinicopathologic and demographic variables, including more advanced primary tumor or nodal stage, HER2-positivity, younger age, and premenopausal status^10–14^. However, it is not currently possible to reliably predict post-pCR recurrence. Prospective identification of patients remaining at high risk despite pCR could enable targeted use of intensified post-neoadjuvant treatment and/or more stringent monitoring approaches.

It is clear that the molecular and immunologic characteristics of the breast tumor and its microenvironment are important determinants of both treatment response and prognosis^7^. In the neoadjuvant setting, pCR attainment is predicted by the degree of tumor lymphocyte infiltration^15^, as well as tumor transcriptomic features including intrinsic subtype and expression of genes involved in proliferation, immune regulation, and cell signaling^7,16–18^. However, there are only limited data regarding the impact of such parameters on the risk of relapse in the context of pCR^19^.

With an intention of identifying transcriptomic changes associated with post-pCR recurrence, we compared the expression of an extensive panel of genes and gene signatures in matched primary and recurrent tumors from the same cohort of patients from our institutional database of >4500 breast cancer patients whose primary tumors had achieved pCR (intraindividual comparison). In addition, we also assessed differential gene expression between the primary tumors from these patients and the primary tumors from matched controls with pCR who did not relapse (interindividual comparison).

## Results

### Patient population

**Fig. 1:**
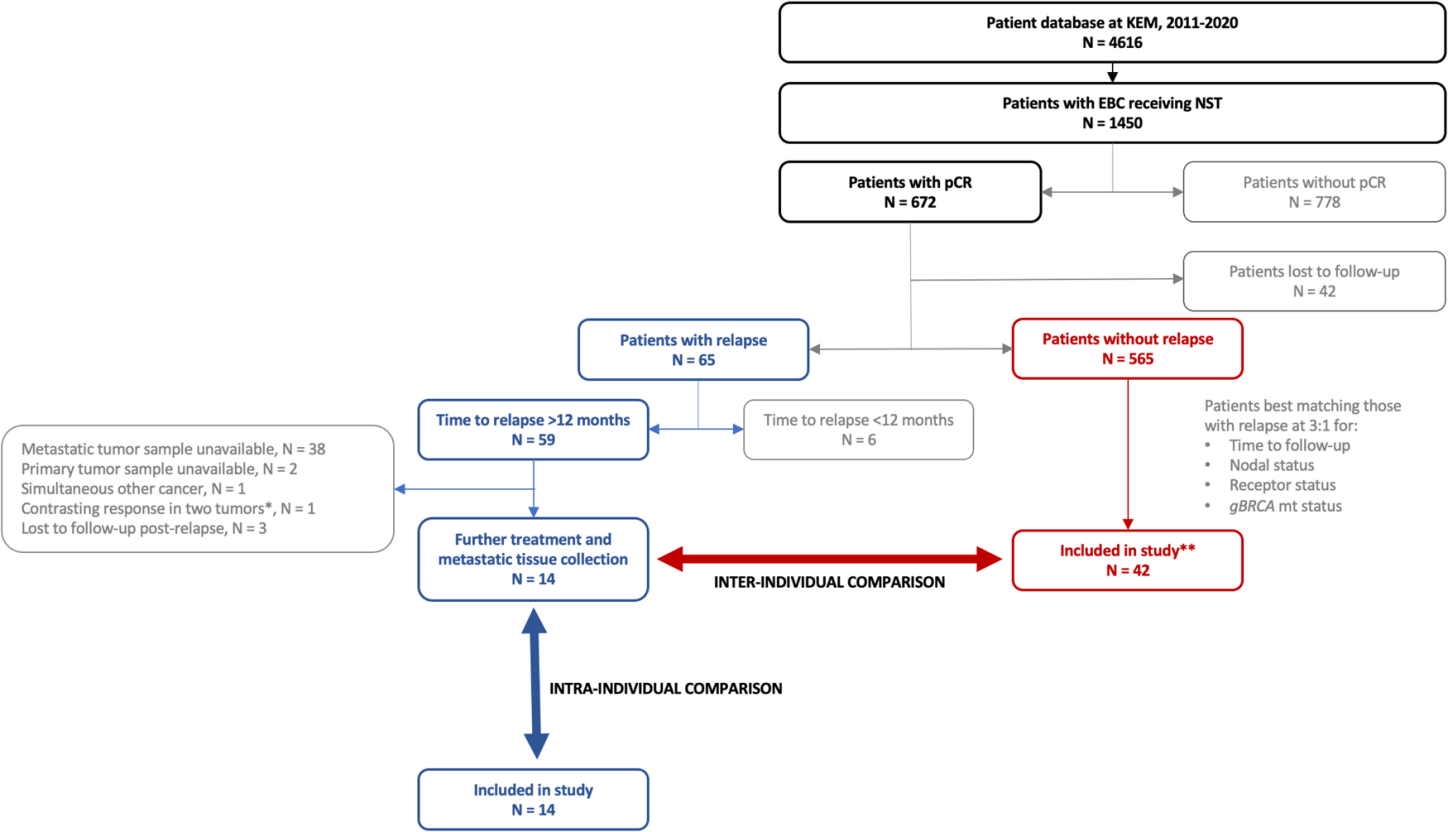
Flowchart identifying patient and control selection. *Two primaries in the same breast, one relapsed while the other showed continued pCR. **A total of 42 controls were identified but immunohistochemical analysis failed in one control. Abbreviations: EBC early breast cancer, KEM Kliniken Essen-Mitte, NST neoadjuvant systemic therapy, pCR pathologic complete response.

From a total of 4616 primary breast cancer patients in our database we identified 1450 EBC patients who had received NST prior to surgery (Fig. 1). The tumors in approximately half of these patients (*n* = 672, 46.3%) demonstrated pCR regardless of the breast cancer subtype. The rate of relapse in patients whose tumors experienced pCR was 9.7% (*n*/*N* = 65/672). After further shortlisting of patients as described in the “Methods” section, tumor samples from a total of 14 patients (primary and recurrent tumor) and 41 matched controls (primary tumor) were sent to NanoString Technologies Germany GmbH (Hamburg, Germany) for transcriptomic analysis using the BC360 panel.

Patient and control characteristics are described in Table 1. In both groups, the majority were node-positive and presented with grade 3, stage T1–T2 tumors at the time of diagnosis. According to immunohistochemical analysis, these tumors were classified as—HR-positive HER2-negative (*n* = 4, 28.6%), HER2-positive (*n* = 5, 35.7%), and TNBC (*n* = 5, 35.7%) (Table 1). In patients whose tumors had g*BRCA1* mt status (*n* = 2), the primary tumors were estrogen receptor (ER)-low positive (<10%)/progesterone receptor (PR)-negative in one case and ER-negative/PR-positive in the other.

**Table 1:** Patient characteristics.

	Patients with relapse despite pCR (*N* = 14)	Controls with pCR and no relapse (*N* = 41)
Median (range) age, years	47.5 (24–73)	49 (32–78)
*Nodal involvement*^*a*^*, n (%)*		
Node-positive	8 (57.1)	23 (56.1)
Node-negative	6 (42.9)	18 (43.9)
*Tumor stage*^*a*^*, n (%)*		
T1	7 (50.0)	16 (39.0)
T2	5 (35.7)	20 (48.8)
T3	1 (7.1)	3 (7.3)
T4	1 (7.1)	2 (4.9)
*Relapse, n (%)*		
Local	6 (42.9)	-
Distant	8 (57.1)	-
*Receptor status, n (%)*		
HR-positive HER2-negative	4 (28.6)^b^	12 (29.3)
HER2-positive	5 (35.7)	15 (36.6)
TNBC^c,d^	5 (35.7)	14 (34.2)^e^
*Grade, n (%)*		
1	–	–
2	2 (14.3)	3 (7.3)
3	12 (85.7)	38 (92.7)
Median (range) Ki-67 index, %	60 (15–90)	50 (10–90)
*Neoadjuvant treatment, n (%)*		
Anthracycline	13 (92.9)	37 (90.2)
Taxane	14 (100.0)	41 (100.0)
Carboplatin	6 (42.9)	12 (29.3)
Anti-HER2 directed therapy	5 (35.7)	15 (36.6)
*Adjuvant treatment*^*f*^*, n (%)*		
Endocrine therapy	9 (64.3)	19 (46.3)
Anti-HER2-therapy	5 (35.7)	15 (36.6)

^a^Data on nodal involvement and tumor stage are pre-NST.

^b^Includes two patients with *gBRCA* mt. Primary tumor of one patient was ER-low PR-negative and of the other patient was ER-negative PR-positive and hence assigned HR-positive as per American Society of Clinical Oncology guidelines.

^c^Only two controls were available for a patient with triple-negative breast cancer and distant recurrence.

^d^Gene expression analysis failed for a control matched to a patient with triple-negative breast cancer and local recurrence.

^e^Immunohistochemical evaluation failed for one control matched to a patient with triple-negative breast cancer.

^f^Groups not exclusive, i.e., patients could have received both treatment regimens.

Abbreviations: *ER* estrogen receptor, *gBRCA1* germline *BRCA1,* *HER2* human epidermal growth factor receptor 2, *HR* hormone receptor, *mt* mutation, *pCR* pathologic complete response, *PR* progesterone receptor, *TNBC* triple-negative breast cancer.

Median time from diagnosis to any relapse was 23.5 months (range: 9.0–75.0). The most common sites of recurrence were lymph nodes (regional and distant; *n* = 7; 50.0%), breast (*n* = 6; 42.9%), brain (*n* = 4, 28.6%), liver (*n* = 4, 28.6%), and lung (*n* = 4, 28.6%). A total of eight patients (57.1%) relapsed with distant metastases.

Since only 2 (instead of 3, as was the case for others) matched controls were available for one of the patients with relapse (who had TNBC), the control cohort comprised tumors from 41 patients with pCR and no relapse. Matched controls for the two relapsed patients whose tumors were g*BRCA1* mt included four patients with tumors that had TNBC/g*BRCA1* wt status, due to limited availability of non-relapsed g*BRCA1* mt controls and the clinical similarity between TNBC and *gBRCA* mt.

### Gene expression analysis

A total of 69 RNA samples were analyzed: 14 primary and 14 recurrent tumors from patients with post-pCR relapse, and 41 primary tumors from controls without relapse. Gene expression analysis failed to meet quality control criteria for one sample (a primary tumor from a control matched to a patient with TNBC/g*BRCA1* wt tumor and local recurrence only), providing available data for 68 tumor samples (98.6%). Subgroup assessments for patients with available gene expression data are indicated in Table 1.

### Intrinsic subtype analyses

**Fig. 2:**
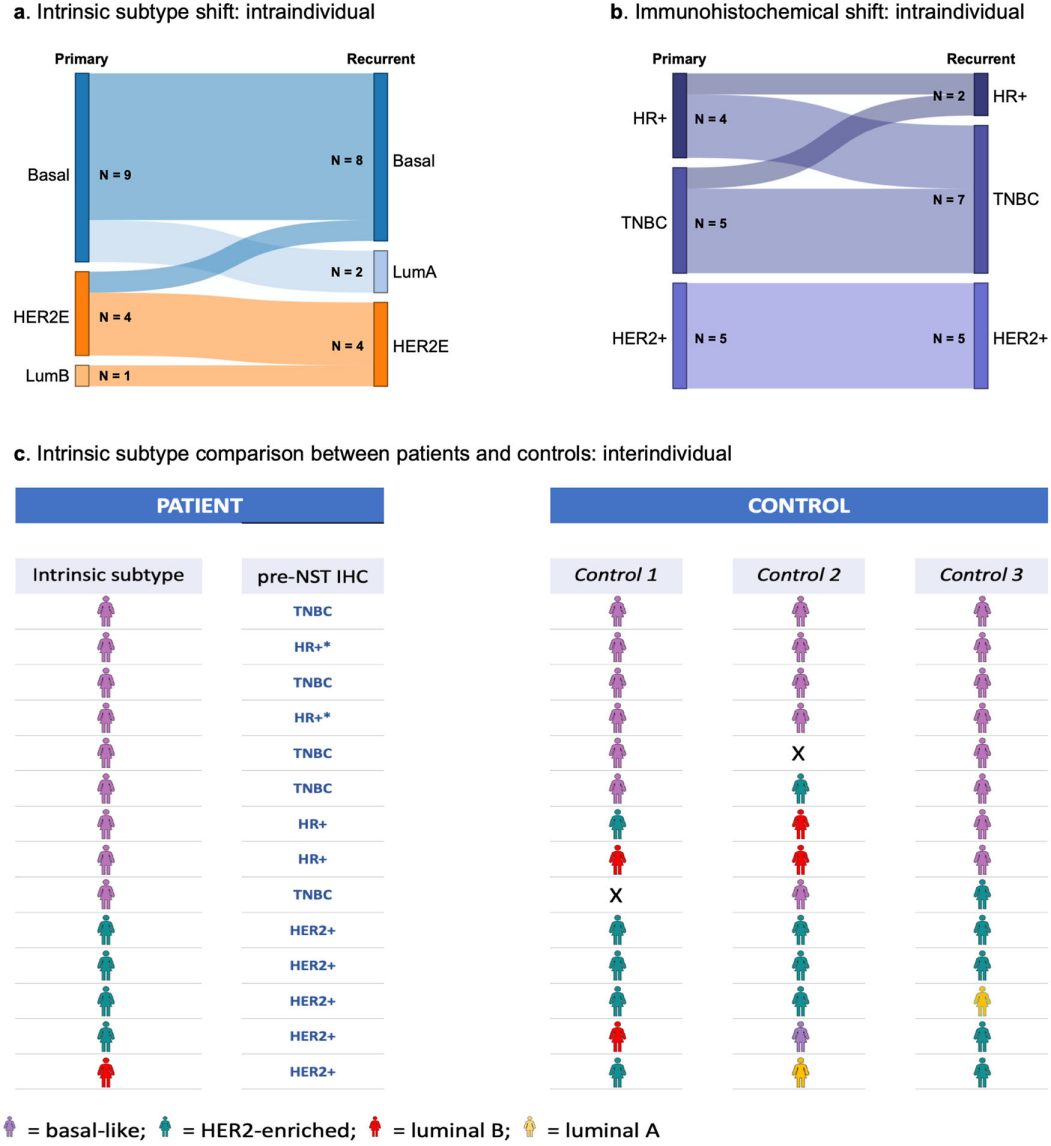
Comparison of intrinsic subtype and immunohistochemistry between patients and controls. Shift in intrinsic subtype (**a**) and immunohistochemical classification (**b**) in between primary tumor and relapse in patients and comparison of intrinsic subtype of primary tumors between patients and matched controls (**c**). For interindividual analysis, patients and controls were matched by immunohistochemistry. *Also *gBRCA1* mt. Abbreviations: HER2 human epidermal growth factor receptor 2, HER2E HER2 expressing, HR hormone receptor, IHC immunohistochemistry, LumA luminal A, LumB luminal B, NST neoadjuvant systemic therapy, TNBC triple-negative breast cancer.

Intrinsic subtype of patients’ tumors (*N* = 14) according to PAM50 analysis of the BC360 panel was—luminal B in one (7.1%) patient, HER2-enriched in four (28.6%) patients, and basal-like in nine (64.3%) patients. Intrinsic subtype differed between primary and recurrent tumors in four (28.6%) patients (Fig. 2a). Of those, two were TNBC which converted from basal-like primaries to luminal A recurrent tumors. One patient with HER2-positive disease had a HER2-enriched primary tumor and a basal-like recurrence, while one patient with HER2-positive tumor had a luminal B primary and HER2-enriched recurrence. The intrinsic subtype was basal-like for both the primary as well as recurrent tumors in the two patients who were *gBRCA* mt indicating that tumors harboring *gBRCA* mutations behave fundamentally similar to TNBC despite some ER or PR expression. In contrast, comparison of the immunohistochemical analysis of the primary tumors to their corresponding recurrent tumors showed an entirely different pattern (Fig. 2b). In this case, while all patients with HER-positive primary tumors presented with HER-positive recurrences, a substantial proportion of patients with HR-positive primary tumors relapsed with TNBC tumors.

In controls with pCR and no relapse, intrinsic subtype of tumors (*N* = 40) was luminal A in two patients (5.0%), luminal B in four patients (10.0%), HER2-enriched in 14 patients (35.0%) and basal-like in 20 patients (50.0%) (Fig. 2c). As matching was done according to immunohistochemistry (IHC) and nodal status, primary tumors from patients and their matched controls had the same IHC. Primary tumor intrinsic subtype in controls was the same as that of their matched patient in 28/40 cases (70.0%). This disparity can be attributed to matching tumors from patients to those in control using classical IHC analyses prior to intrinsic subtyping using the BC360 panel.

### Interindividual comparison

**Fig. 3:**
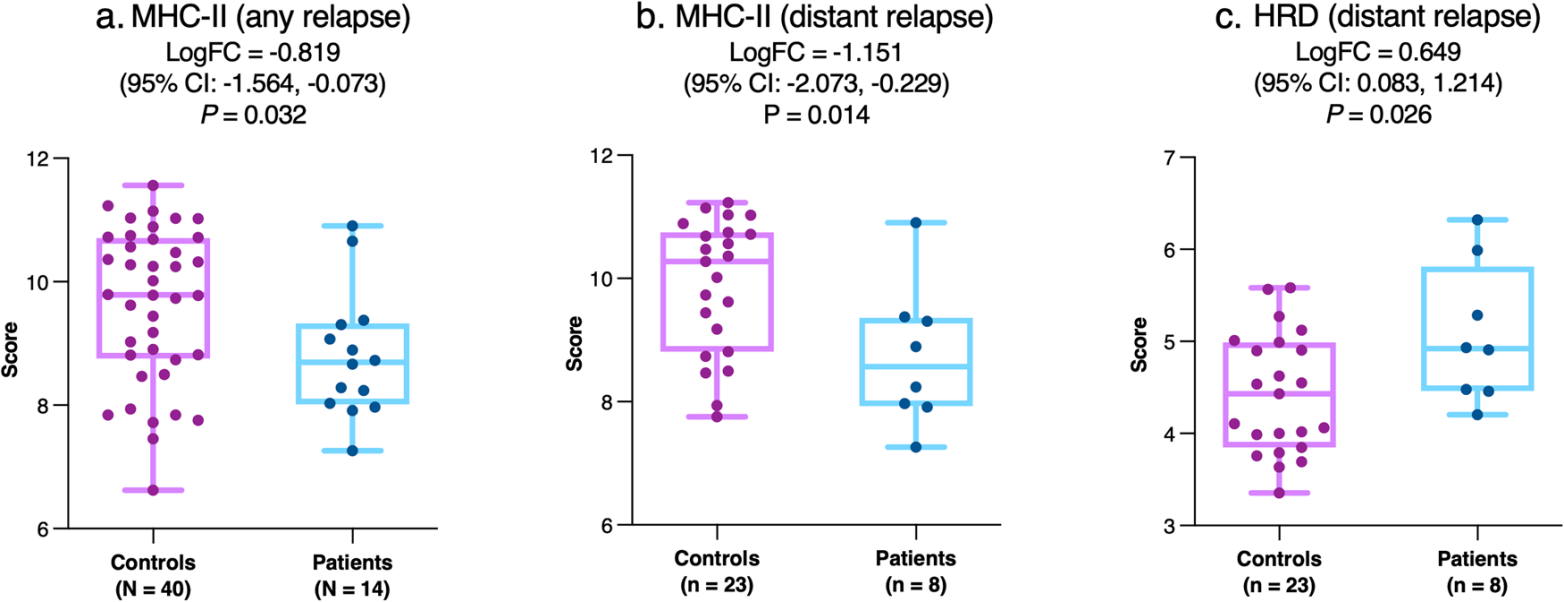
Interindividual comparison of primary tumor gene expression between patients with relapse despite pCR and controls with pCR and no relapse. Data are shown for MHC-II (**a**, **b**) and HRD (**c**) signature expression for the overall cohort (**a**) and for the distant relapse subgroup (**b**, **c**). The central line indicates the median, the box indicates the 25th and 75th percentiles and the whiskers indicate the range. Circles represent individual data points. Negative values for logFC indicate lower expression in patients with relapse versus controls. Abbreviations: CI confidence interval, HRD homologous recombination deficiency; logFC log_2_-fold change, MHC-II major histocompatibility complex-class II.

Primary tumor expression of major histocompatibility complex-class II (MHC-II) molecules was significantly lower in the tumors of patients who later relapsed despite pCR compared with controls (Fig. 3a). This difference appeared more pronounced in tumors from patients with distant relapse (Fig. 3b). Patients with distant relapse also showed a trend for decreased interferon gamma (IFNγ) signaling (logFC = −0.759; 95% confidence interval (CI): −1.534 to 0.172; *P* = 0.055) and significantly higher homologous recombination deficiency (HRD) signature expression in the primary tumor versus controls (Fig. 3c; Supplementary Table S1).

**Fig. 4:**
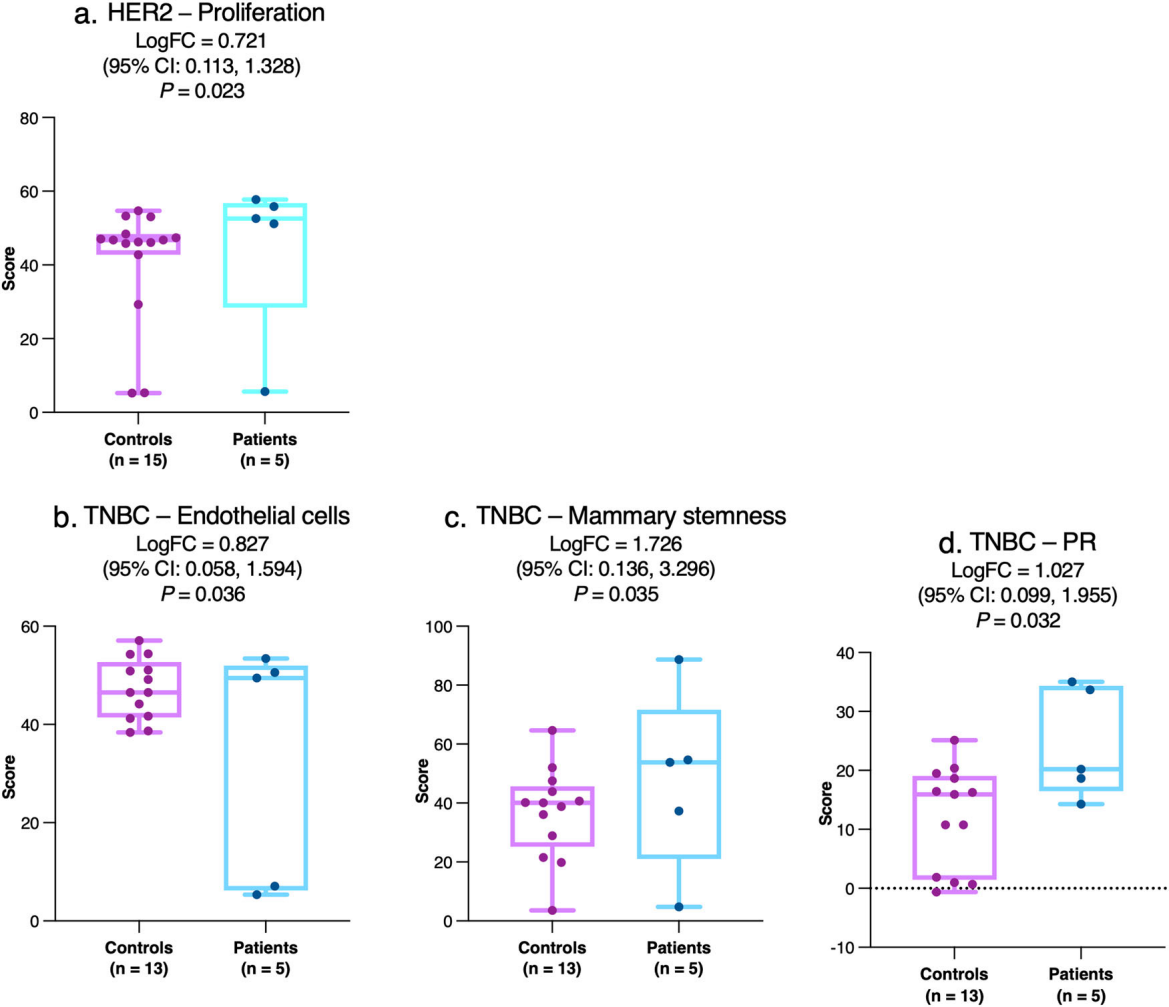
Interindividual comparison of primary tumor gene expression between patients and matched controls with HER2-positive disease or TNBC. For the HER2 subgroup, data are shown for proliferation (**a**) while for the TNBC subgroup, data are shown for endothelial cell activation (**b**), mammary stemness (**c**), and progesterone receptor (**d**). The central line indicates the median, the box indicates the 25th and 75th percentiles and the whiskers indicate the range. Circles represent individual data points. Negative values for logFC indicate lower expression in patients with relapse versus controls. Abbreviations: CI confidence interval, HER2 human epidermal growth factor receptor 2, logFC log_2_-fold change, PR progesterone receptor, TNBC triple-negative breast cancer.

Several other differences were observed between primary tumors from patients with any relapse despite pCR and controls in subgroup analyses. In patients with HER2-positive tumors, proliferation score was significantly higher versus controls (Fig. 4a). In patients with TNBC and relapse, tumor expression of endothelial cell signature, mammary stemness signature, and PR gene was significantly greater than in controls (Fig. 4b–d). *P* values in interindividual analyses were not significant after false detection rate (FDR) adjustment (Supplementary Tables S1 and S2).

### Intraindividual comparison

**Fig. 5:**
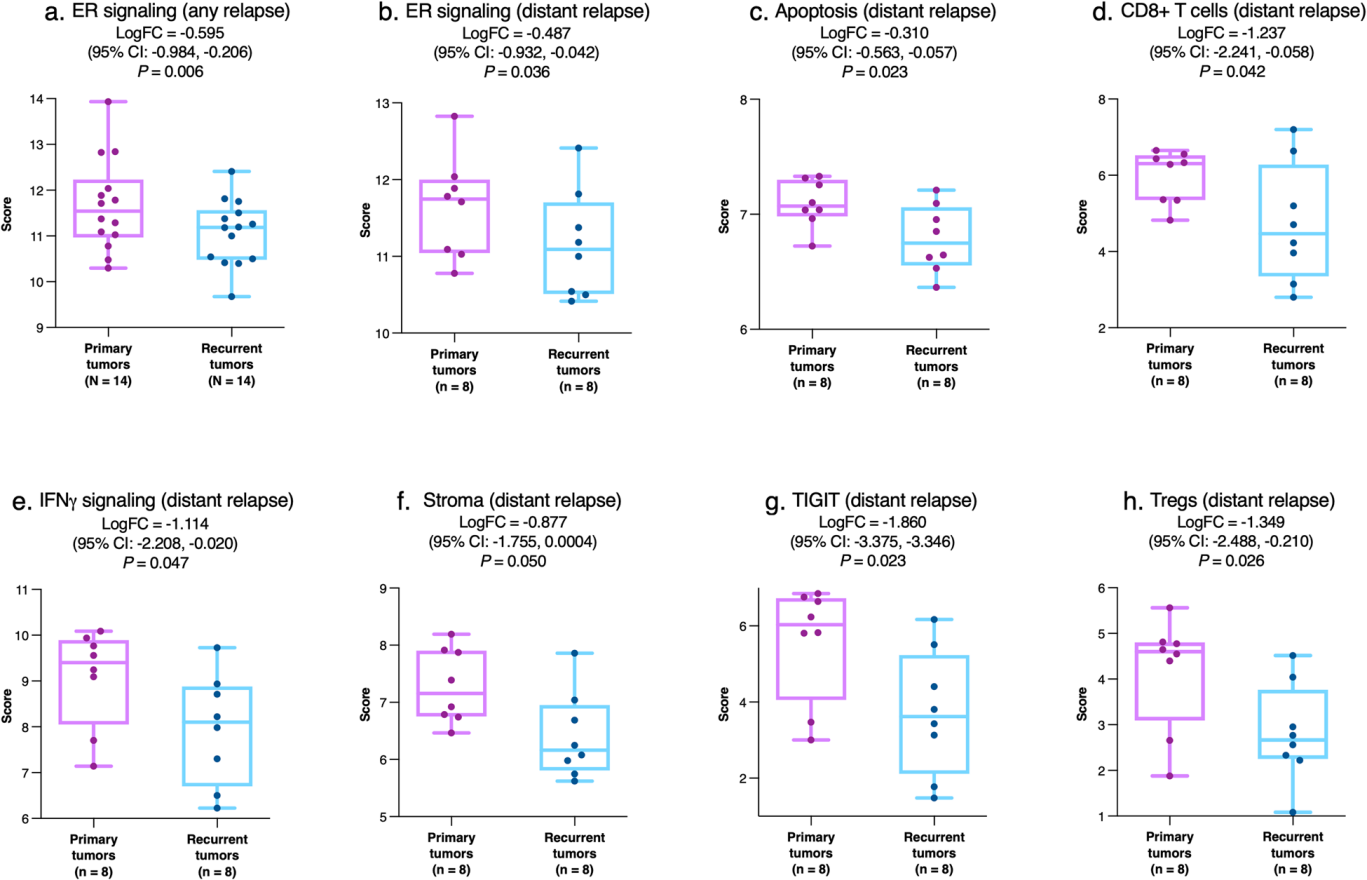
Intraindividual comparison of gene expression between matched primary tumors and post-pCR recurrences. Data are shown for ER signaling (**a**, **b**), apoptosis (**c**), CD8+ T cells (**d**), IFNγ signaling (**e**), stroma (**f**), TIGIT (**g**), and Treg (**h**) signature expression for the overall cohort (**a**) and for the distant relapse subgroup (**b**–**h**). The central line indicates the median, the box indicates the 25th and 75th percentiles and the whiskers indicate the range. Circles represent individual data points. Negative values for logFC indicate lower expression in recurrent versus primary tumors. Abbreviations: CI confidence interval, ER estrogen receptor, IFN-γ interferon gamma, logFC log_2_-fold change, TIGIT T-cell immunoreceptor with Ig and ITIM domains, Tregs regulatory T-cells.

Compared with primary tumors, post-pCR recurrences had significant downregulation of ER signaling signature expression (Fig. 5a). This effect was also seen when the analysis was restricted to distant recurrences (Fig. 5b), which additionally showed lower expression of genes or signatures for apoptosis, CD8+ T-cells, IFNγ signaling, stromal cells, T-cell immunoreceptor with immunoglobulin and immunoreceptor tyrosine-based inhibition motif domains (TIGIT), and regulatory T cells (T_reg_), relative to the primary tumor (Fig. 5c–h; Supplementary Table S3).

**Fig. 6:**
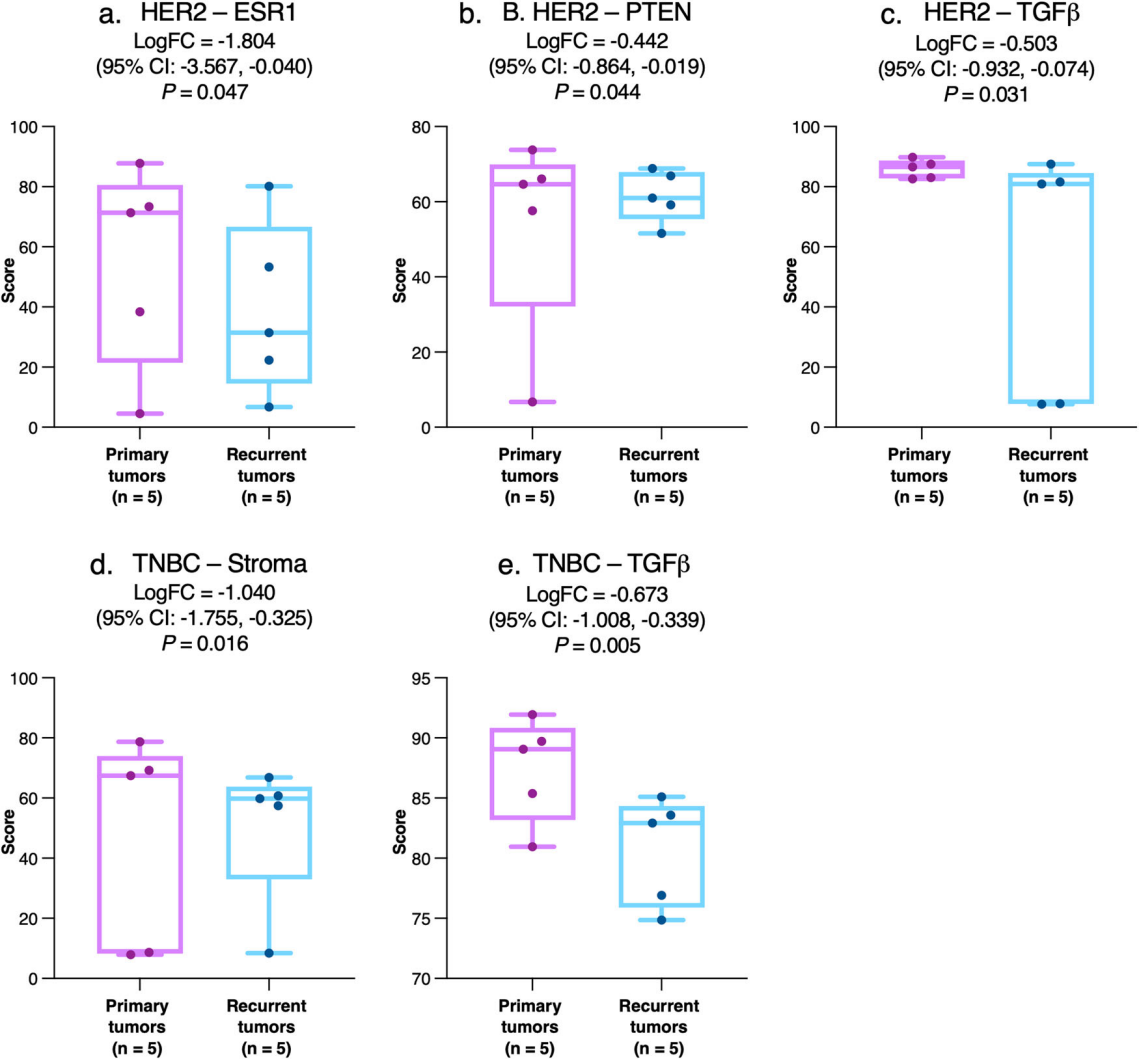
Intraindividual comparison of tumor gene expression between matched primary tumors and post-pCR recurrences that were HER2-positive or TNBC. For the HER2 subgroup, data are shown for estrogen signaling (**a**), PTEN (**b**), and TGFβ (**c**) while for the TNBC subgroup, data are shown for stromal signaling (**d**), and TGFβ (**e**). The central line indicates the median, the box indicates the 25^th^ and 75^th^ percentiles and the whiskers indicate the range. Circles represent individual data points. Negative values for logFC indicate lower expression in patients with relapse versus controls. Abbreviations: CI confidence interval, ESR1 estrogen receptor 1, logFC log_2_-fold change, PTEN phosphatase and tensin homolog, TGFß transforming growth factor beta, TNBC triple-negative breast cancer.

Significant differences were also observed in subgroup analyses conducted irrespective of the site(s) of recurrence according to the pathologic classification of the primary tumor. *ESR1* was downregulated in recurrent tumors in the HER2-positive subgroup (Fig. 6a), along with decreases in genes coding the phosphatase and tensin homolog, a tumor suppressor (Fig. 6b) and transforming growth factor-β (TGFβ) (Fig. 6c), a multifunctional cytokine. In the TNBC subgroup, expression of both TGFβ and the stromal cell signature were downregulated in the recurrent tumor (Fig. 6d, e). *P* values in intraindividual analyses were not significant after FDR adjustment (Supplementary Tables S3 and S4).

## Discussion

Despite a strong favorable prognosis following pCR, there is an unmet need for biomarkers to identify the substantial minority of EBC patients who are still at a risk of relapse. To our knowledge, ours is the first such study investigating the association between gene expression with both tumor evolution at relapse (intraindividual analysis) and relapse risk (interindividual analysis). Using a transcriptomic approach, our analysis of >4500 patients with primary breast cancer in our institute’s database, of whom approximately 10% experienced relapse post pCR, was able detect differential expression of several key pathways mediating tumor biology and progression such as antitumor immunity, DNA damage response, stromal factors, hormonal signaling, and tumor regulation.

The interaction between the breast tumor and the immune system plays a key role in shaping the course and outcome of the disease^20^. MHC-II is an important driver of immune activation which further leads to an increase in tumor response^21–24^. Various studies have identified a correlation between lowered MHC-II expression and reduced tumor lymphocyte infiltration, greater lymphovascular invasion, and poor outcome in patients with TNBC treated with or without adjuvant chemotherapy^23–25^. Attenuated MHC-II expression is expected to limit activation of CD4+ T cells, which mediate anticancer immunity by facilitating CD8+ T cell activation, secreting effector cytokines and direct cell-killing^26^, and also regulate metastasis via effects on the vasculature^27^. Although predominantly expressed by professional antigen-presenting cells, MHC-II can be induced in breast cancer cells by IFNγ released by activated T cells into the microenvironment^28^. Our interindividual analyses demonstrated a lower expression of MHC-II in the primary tumors of patients with post-pCR relapse in comparison with those from their matched controls (Fig. 3a, b). Furthermore, the reduced MHC-II expression was paralleled by a trend for lower expression of the IFNγ signaling signature, albeit only in the distant relapse subgroup. Interestingly, tumors may adapt to suppress IFNγ-mediated induction of MHC-II by activation of the Ras-mitogen-activated-protein-kinase (RAS/MAPK) pathway, which is implicated in immune evasion of residual TNBC persisting after NST^29^.

Another signal of interest we report from our interindividual analyses is the substantially higher expression of HRD signature (from the 143 DNA damage repair genes in the BC360 panel) in primary tumors from patients with post-pCR relapse in comparison to those from the controls (Fig. 3c). The role of HRD, especially in TNBC, has been explored widely in recent times, from a therapeutic as well as a prognostic angle. While the former has led to the discovery of a number of poly-adenosinediphosphateribose polymerase (PARP) inhibitors for clinical use in metastatic and advanced breast cancer^30^, the importance of HRD assessment for predicting treatment response as well as attainment of pCR is also currently being elucidated. Although it is common knowledge that the homologous recombination DNA repair pathway harbors mutations in large proportion of TNBCs, Timms et al were the first to postulate that other breast cancer subtypes could also present defects in this pathway^31^ and hence a metric of HRD could be utilized for clinical assessment of treatment outcomes. A recent comparison of *BRCA*-positive tumors to *BRCA*-negative tumors also showed a higher HRD score in the former^32^. From a translational perspective, HRD status has also been shown to be predictive of achievement of pCR in patients with TNBC/*BRCA*-positive breast cancer^33^ as well as those with HR-positive breast cancer^34^.

Our interindividual analysis demonstrated higher primary tumor expression of endothelial cell and mammary stemness signatures than controls in patients with TNBC who subsequently relapsed (Fig. 4b, c). Detected by immunohistochemistry, both endothelial cells (reflected in microvessel density) and cancer stem cells are most abundant in TNBC and are poor prognostic factors^35–37^. Cancer stem cells, in particular, are critical determinants of metastatic dissemination and treatment resistance, as underscored by their enrichment in occult metastatic lesions and residual disease post-therapy^38^. In HER2-positive tumors we saw an association of higher pretherapeutic proliferation index with post-pCR recurrence. Greater baseline tumor proliferation, as reflected in Ki-67 positivity, has previously been associated with higher pCR rates across breast cancer subtypes, but its prognostic significance in the neoadjuvant setting may be dependent not only on subtype but also on pCR status^39^.

We observed significant downregulation of ER signaling signature in post-pCR recurrences versus paired primary tumors (Fig. 5a, b), with a reduction in the expression of *ESR1* in HER2-positive subgroups (Fig. 6a). Loss or mitigation of ER signaling is a known mechanism of endocrine resistance occurring in around one-fifth of initially ER-positive tumors and may be accompanied by the upregulation of alternative growth-stimulating pathways^40^. We also noted a paradoxical association between tumor PR signature and relapse in patients who had TNBC and therefore negative PR expression by immunohistochemistry. This finding likely reflects differences in PR expression at the mRNA and protein level^41^, which could be introduced by translational regulation, for example, by microRNAs^42^.

Taken together, the output of our analyses poses two main clinical implications. Firstly, our results appear to suggest that post-pCR recurrence is associated with a change in tumor immune microenvironment from an anti-tumorigenic to a pro-tumorigenic phenotype. Indeed, through interindividual comparison we have shown the altered expression of certain known drivers of anti-tumorigenic immune activity such as MHC-II in tumors of patients who subsequently developed recurrent tumors. Furthermore, through intraindividual comparison we have shown a downregulation of key anti-tumorigenic effectors of the immune system such as CD8+ T cells (Fig. 5d), IFN-γ (Fig. 5e), TIGIT (Fig. 5g), and T_reg_ cells (Fig. 5h) in recurrent tumors. A number of recent studies postulate that such alterations in the tumor immune microenvironment are in response to NST and are likely to have predictive value in treatment outcomes^43–45^. Secondly, our findings suggest that the immunologically quiescent primary tumors which are at risk of post-pCR relapse could benefit from the early use of immune-oncologic agents either alone or in combination with NST. The improvement in pCR achievement rate in early TNBC from the addition of immune checkpoint inhibitors to NST as seen from certain landmark clinical trials such as IMpassion031^46^, Keynote 522^47^, and I-SPY2^48^ indicate a validity in our suggestion. Consequently, it would be interesting to conduct analyses similar to the one we present here on the datasets of these trials to assess whether early use of immunotherapy is effective in patients with immunologically quiescent tumors who achieve pCR. One open question is to what extent the risk factors for relapse after pCR might differ from more general risk factors for recurrence. Many of the variables associated with relapse in the present study have shown negative prognostic significance in wider populations. Previously reported analysis conducted by the German Breast Group evaluated the impact of classical clinical parameters on relapse and concluded that initial tumor size and nodal status were the only prognostic factors associated with long-term survival^11^. However, it is unclear whether these results are comparable to our findings since the vast majority of patients in our analysis were clinically designated as cT1/2 and clinically node negative thereby representing a relatively early stage breast cancer. In addition, since post-pCR recurrence implies discordant response to NST, mechanisms that increase mutational load and clonal heterogeneity, such as HRD, may be especially expected to drive relapse as seen in this setting.

Our analyses have certain limitations. Due to their nature, we had to retrospectively identify eligible patients and controls from our database and hence had no control over the sample size. Consequently, our subgroup analyses for different breast cancer subtypes were underpowered. Moreover, given the relatively short time to recurrence, we could not gather certain types such as luminal A breast cancers which are known to relapse much later. In addition, our analysis was based on data from a single center, and this could affect generalizability of our findings. Finally, statistical significance did not persist after FDR adjustment.

A key strength of our study was the requirement for time to distant relapse of >12 months, which enabled us to exclude patients with occult primary metastatic disease, who represent a distinct population. In addition, given our institutional practice of collecting tumor tissue for biopsy from the primary as well as relapsed tumor, we could perform paired intraindividual analyses, an advantage not always available to large national and international study groups. We also included both local recurrences and distant metastases since invasive DFS is one of the prime endpoints in breast cancer clinical trials. Taken together, our unique approach of intra- and interindividual analyses allowed us to identify significant differences in several gene expression variables which may drive relapse after pCR attainment.

In conclusion, we have identified several transcriptomic correlates of relapse despite pCR and changes in tumor gene expression associated with recurrences occurring after pCR attainment, which warrant further investigation in prospective trials. Even in patients whose tumors attain a pCR, insufficiently activated immunogenic pathways may play a key role for relapse. If validated, the identified risk factors and mechanisms of recurrence could inform the design of novel post-neoadjuvant strategies, for patients who are at a risk of recurrence despite their tumors achieving pCR^8,9^. For example, tumors with HRD may be sensitive to platinum agents and PARP inhibitors^49,50^. Moreover, the role of immune oncology in the neoadjuvant setting is still under investigation and genomic analysis of patients after NST comprising immuno-oncological agents may play a role in future to identify patients who benefit most from tailored therapy strategies. Conversely, downregulation of immune markers in post-pCR recurrences suggests that immunotherapies may be better deployed earlier in the course of disease^51^. Given the absence of tumor in surgical specimens at pCR, liquid biopsy methods for quantifying residual disease burden and tracking tumor evolution after NST particularly holds a promise for the future personalization of therapy in this setting^52^.

## Patients and methods

### Study design and patients

This study involved a retrospective analysis of female patients diagnosed with early or locally advanced breast cancer at a single center (Kliniken Essen-Mitte [KEM]) in Essen, Germany between September 2011 and January 2020.

In order to identify appropriate study patients and controls, we evaluated the records of breast cancer patients in KEM’s database for the aforementioned period (Fig. 1). Data eligible for analysis came from patients who had—received standard of care NST for a minimum of 12 weeks followed by requisite surgery to the breast and the axillary lymph nodes; had subsequently attained pCR, as defined by absence of invasive cancer in the breast and axilla (i.e., ypT0/is and ypN0); had received appropriate treatment and follow-up on relapse; and had biopsy samples from the primary cancer (acquired prior to initiating NST) as well as from the site of recurrence available. Certain patients in whom further treatment was deemed not necessary by the treating physician, e.g., those with TNBC achieving pCR following mastectomy, were also considered eligible for inclusion in our analysis. To exclude occult primary metastatic disease, we discounted patients in whom the time to distant relapse was <12 months.

Eligible controls were patients in our database who were relapse-free following pCR for a period comparable to the corresponding study patient and also matched them for receptor status (HR, HER), nodal status, or *gBRCA* status using baseline immunohistochemical analysis. For patients with g*BRCA1* mutation (mt) status, if no matching g*BRCA1* mt control was available, the patient was instead matched to a control with TNBC and g*BRCA1* wildtype (wt) status. We aimed to identify three controls for every patient who fulfilled the aforementioned criteria.

Ethics committee approval was obtained from the institutional ethics committee (Ethik-Kommission der Ärztekammer Nordrhein, Düsseldorf, Germany) and patients had provided written informed consent previously.

### Sample processing and gene expression analysis

Biopsy specimens of patients (primary as well as recurrent tumors) and controls (primary tumors), previously preserved by fixing in formalin and embedding in paraffin according to KEM’s standard protocol were retrieved and sent to NanoString Technologies Germany GmbH (Hamburg, Germany) for analysis using the Breast Cancel 360 (BC360) panel on the multiplexed digital nCounter^®^ platform (NanoString Technologies Inc., Seattle, WA, USA).

Briefly, RNA from biopsy specimens were isolated and when necessary, amplified, using NanoString’s in-house standardized protocol, followed by hybridization with the BC360 panel and a signal readout on the nCounter^®^ System.

The BC360 panel consists of 758 genes of interest for breast tumor biology, including the 50-gene prediction analysis of microarray (PAM50) set^53,54^, and 18 housekeeping control genes^55^. Gene counts were normalized to housekeeping gene expression, as well as either a panel standard (for non-PAM50 genes) or a reference sample (for PAM50 genes). Normalized gene expression data were log_2_-transformed and used to derive expression scores for the 42 genes and gene signatures that are a preselected focus of the panel and which reflect tumor biology, the immune response and abundance of different cell populations in the microenvironment. The PAM50 gene set enabled determination of the intrinsic subtype (i.e., luminal A, luminal B, HER2-enriched or basal-like) and risk of recurrence score according to published methods^53,54^.

### Statistics

Since our analysis entailed identifying eligible patients and controls from our institutional database, no formal sample size calculation was performed. The decision to identify three controls to every patient for interindividual analysis was also arbitrary although we attempted to stringently match patients and controls according to their immunohistochemical profile.

Differential gene expression was expressed as log_2_-fold change (logFC) with its associated 95% CI. Statistical significance was assessed using Student’s *t*-test (paired for intraindividual comparison and unpaired for interindividual comparison). *P* values were corrected for multiplicity using Benjamini–Yekutieli false-discovery rate (FDR) adjustment^56^. Subgroup analyses were performed to analyze differences between any and distant relapse or those between tumor categorized by immunohistochemistry (HR-positive/HER2-negative; HER2-positive [irrespective of HR status]; TNBC); and g*BRCA1* status. For subgroups in interindividual analyses, controls were included based on the characteristics of their matched patient.

Analyses were performed using SPSS Statistics Version 23.0 (IBM Corporation, Armonk, NY, USA) and Prism Version 9.0 (GraphPad Software Inc., San Diego, CA, USA).

### Reporting summary

Further information on research design is available in the Nature Research Reporting Summary linked to this article.

## Supplementary information

Supplementary Tables

Reporting summary checklist from NPJ

## Data Availability

Data supporting the findings in this study in Table 1 and Figs. 2–6 as well as Supplementary Tables S1–S4 are available.

## References

[1] Cardoso, F. et al. Early breast cancer: ESMO Clinical Practice Guidelines for diagnosis, treatment and follow-up. *Ann. Oncol*. 10.1093/annonc/mdz173 (2019). 10.1093/annonc/mdz189 31236598

[2] Hayes, D. F. & Schott, A. F. Neoadjuvant chemotherapy: What are the benefits for the patient and for the investigator? *J. Natl Cancer Inst.*10.1093/jncimonographs/lgv004 (2015). 10.1093/jncimonographs/lgv004 26063884

[3] Mamtani, A. et al. How often does neoadjuvant chemotherapy avoid axillary dissection in patients with histologically confirmed nodal metastases? Results of a prospective study. *Ann. Surg. Oncol*. 10.1245/s10434-016-5246-8 (2016). 10.1245/s10434-016-5246-8 PMC5070651 27160528

[4] Colomer, R. et al. Neoadjuvant management of early breast cancer: a clinical and investigational position statement. *Oncologist*10.1634/theoncologist.2018-0228 (2019). 10.1634/theoncologist.2018-0228 PMC6516119 30710068

[5] Spring LM (2020). Pathologic complete response after neoadjuvant chemotherapy and impact on breast cancer recurrence and survival: a comprehensive meta-analysis. Clin. Cancer Res..

[6] Cortazar, P. et al. Pathological complete response and long-term clinical benefit in breast cancer: The CTNeoBC pooled analysis. *Lancet*10.1016/S0140-6736(13)62422-8 (2014). 10.1016/S0140-6736(13)62422-8 24529560

[7] Chica-Parrado, M. R. et al. Resistance to neoadjuvant treatment in breast cancer: clinicopathological and molecular predictors. *Cancers*10.3390/cancers12082012 (2020). 10.3390/cancers12082012 PMC7463925 32708049

[8] Masuda, N. et al. Adjuvant capecitabine for breast cancer after preoperative chemotherapy. *N. Engl. J. Med*. 10.1056/NEJMoa1612645 (2017). 10.1056/NEJMoa1612645 28564564

[9] Von Minckwitz, G. et al. Trastuzumab emtansine for residual invasive HER2-positive breast cancer. *N. Engl. J. Med*. 10.1056/NEJMoa1814017 (2019). 10.1056/NEJMoa1814017 30516102

[10] Tanioka, M. et al. Predictors of recurrence in breast cancer patients with a pathologic complete response after neoadjuvant chemotherapy. *Br. J. Cancer*10.1038/sj.bjc.6605769 (2010). 10.1038/sj.bjc.6605769 PMC2920023 20606681

[11] Huober, J. et al. Abstract P2-08-01: Factors predicting relapse in early breast cancer patients with a pathological complete response after neoadjuvant therapy—results of a pooled analysis based on the GBG meta-database. *Cancer Res*. **79** P2-08-01–P2-08–01 (2019).

[12] Chaudry M (2015). Recurrence and survival among breast cancer patients achieving a pathological complete response to neoadjuvant chemotherapy. Breast Cancer Res. Treat..

[13] Asaoka M (2019). Clinical and pathological predictors of recurrence in breast cancer patients achieving pathological complete response to neoadjuvant chemotherapy. Eur. J. Surg. Oncol..

[14] Weiss, A., Bashour, S. I., Hess, K., Thompson, A. M. & Ibrahim, N. K. Effect of neoadjuvant chemotherapy regimen on relapse-free survival among patients with breast cancer achieving a pathologic complete response: an early step in the de-escalation of neoadjuvant chemotherapy. *Breast Cancer Res*. 10.1186/s13058-018-0945-7 (2018). 10.1186/s13058-018-0945-7 PMC5902970 29661243

[15] Denkert C (2018). Tumour-infiltrating lymphocytes and prognosis in different subtypes of breast cancer: a pooled analysis of 3771 patients treated with neoadjuvant therapy. Lancet Oncol..

[16] Prat A (2016). Prediction of response to neoadjuvant chemotherapy using core needle biopsy samples with the prosigna assay. Clin. Cancer Res..

[17] Callari, M. et al. Subtype-specific metagene-based prediction of outcome after neoadjuvant and adjuvant treatment in breast cancer. *Clin. Cancer Res*. 10.1158/1078-0432.CCR-15-0757 (2016). 10.1158/1078-0432.CCR-15-0757 26423797

[18] Esserman LJ (2012). Chemotherapy response and recurrence-free survival in Neoadjuvant breast cancer depends on biomarker profiles: results from the I-SPY 1 TRIAL (CALGB 150007/150012; ACRIN 6657). Breast Cancer Res. Treat..

[19] Takeshita T (2020). Transcriptomic and functional pathway features were associated with survival after pathological complete response to neoadjuvant chemotherapy in breast cancer. Am. J. Cancer Res..

[20] Annaratone L (2020). The multifaceted nature of tumor microenvironment in breast carcinomas. Pathobiology.

[21] Wulfkuhle JD (2019). Quantitative MHC II protein expression levels in tumor epithelium to predict response to the PD1 inhibitor pembrolizumab in the I-SPY 2 Trial. J. Clin. Oncol..

[22] Oldford SA (2006). Tumor cell expression of HLA-DM associates with a Th1 profile and predicts improved survival in breast carcinoma patients. Int. Immunol..

[23] Andres, F. et al. Expression of the MHC class II pathway in triple-negative breast cancer tumor cells is associated with a good prognosis and infiltrating lymphocytes. *Cancer Immunol. Res*. 10.1158/2326-6066.CIR-15-0243 (2016). 10.1158/2326-6066.CIR-15-0243 PMC4878913 26980599

[24] Park, I. A. et al. Expression of the MHC class II in triple-negative breast cancer is associated with tumor-infiltrating lymphocytes and interferon signaling. *PLoS ONE*10.1371/journal.pone.0182786 (2017). 10.1371/journal.pone.0182786 PMC5560630 28817603

[25] Stewart, R. L. et al. A multigene assay determines risk of recurrence in patients with triple-negative breast cancer. *Cancer Res*. 10.1158/0008-5472.CAN-18-3014 (2019). 10.1158/0008-5472.CAN-18-3014 PMC6629024 31048497

[26] Tay, R. E., Richardson, E. K. & Toh, H. C. Revisiting the role of CD4+ T cells in cancer immunotherapy—new insights into old paradigms. *Cancer Gene Therapy*10.1038/s41417-020-0183-x (2020). 10.1038/s41417-020-0183-x PMC7886651 32457487

[27] Blomberg, O. S., Spagnuolo, L. & De Visser, K. E. Immune regulation of metastasis: mechanistic insights and therapeutic opportunities. *DMM Disease Models and Mechanisms* (2018) 10.1242/dmm.036236. 10.1242/dmm.036236 PMC6215427 30355585

[28] Axelrod, M. L., Cook, R. S., Johnson, D. B. & Balko, J. M. Biological consequences of MHC-II expression by tumor cells in cancer. *Clin. Cancer Res.*10.1158/1078-0432.CCR-18-3200 (2019). 10.1158/1078-0432.CCR-18-3200 PMC6467754 30463850

[29] Loi, S. et al. RAS/MAPK activation is associated with reduced tumor-infiltrating lymphocytes in triple-negative breast cancer: therapeutic cooperation between MEK and PD-1/PD-L1 immune checkpoint inhibitors. *Clin. Cancer Res*. 10.1158/1078-0432.CCR-15-1125 (2016). 10.1158/1078-0432.CCR-15-1125 PMC4794351 26515496

[30] Cortesi L, Rugo HS, Jackisch C (2021). An overview of PARP inhibitors for the treatment of breast cancer. Target. Oncol..

[31] Timms KM (2014). Association of BRCA1/2defects with genomic scores predictive of DNA damage repair deficiency among breast cancer subtypes. Breast Cancer Res.

[32] Force, J. et al. Abstract P3-08-07: distinct biological signatures describe differences in BRCA mutated subgroups. *Cancer Res*. **79**, P3-08–07 (2019).

[33] ML T (2018). Homologous recombination deficiency (HRD) status predicts response to standard neoadjuvant chemotherapy in patients with triple-negative or BRCA1/2 mutation-associated breast cancer. Breast Cancer Res. Treat..

[34] C D (2021). Reconstructing tumor history in breast cancer: signatures of mutational processes and response to neoadjuvant chemotherapy ⋆. Ann. Oncol..

[35] Mohammed, R. A. A. et al. Lymphatic and blood vessels in basal and triple-negative breast cancers: characteristics and prognostic significance. *Mod. Pathol*. 10.1038/modpathol.2011.4 (2011). 10.1038/modpathol.2011.4 21378756

[36] Uzzan, B., Nicolas, P., Cucherat, M. & Perret, G. Y. Microvessel density as a prognostic factor in women with breast cancer: a systematic review of the literature and meta-analysis. *Cancer Res.*10.1158/0008-5472.CAN-03-1957 (2004). 10.1158/0008-5472.can-03-1957 15126324

[37] Giatromanolaki, A., Sivridis, E., Fiska, A. & Koukourakis, M. I. The CD44+/CD24- phenotype relates to ‘triple-negative’ state and unfavorable prognosis in breast cancer patients. *Med. Oncol*. 10.1007/s12032-010-9530-3 (2011). 10.1007/s12032-010-9530-3 20405247

[38] Gooding, A. J. & Schiemann, W. P. Epithelial–mesenchymal transition programs and cancer stem cell phenotypes: mediators of breast cancer therapy resistance. *Mol. Cancer Res*. 10.1158/1541-7786.mcr-20-0067 (2020). 10.1158/1541-7786.MCR-20-0067 PMC7483945 32503922

[39] Denkert, C. et al. Ki67 levels as predictive and prognostic parameters in pretherapeutic breast cancer core biopsies: a translational investigation in the neoadjuvant gepartrio trial. *Ann. Oncol*. 10.1093/annonc/mdt350 (2013). 10.1093/annonc/mdt350 23970015

[40] Osborne, C. K. & Schiff, R. Mechanisms of endocrine resistance in breast cancer. *Annu. Rev. Med*. 10.1146/annurev-med-070909-182917 (2011). 10.1146/annurev-med-070909-182917 PMC3656649 20887199

[41] Tong D (1999). Messenger RNA determination of estrogen receptor, progesterone receptor, pS2, and plasminogen activator inhibitor-1 by competitive reverse transcription-polymerase chain reaction in human breast cancer. Clin. Cancer Res..

[42] Gilam, A. et al. MicroRNA regulation of progesterone receptor in breast cancer. *Oncotarget*10.18632/oncotarget.15657 (2017). 10.18632/oncotarget.15657 PMC5432230 28404930

[43] Park YH (2020). Chemotherapy induces dynamic immune responses in breast cancers that impact treatment outcome. Nat. Commun..

[44] Pérez-Pena J (2019). A Transcriptomic immunologic signature predicts favorable outcome in neoadjuvant chemotherapy treated triple negative breast tumors. Front. Immunol.

[45] Graeser M (2021). Original research: Immune cell composition and functional marker dynamics from multiplexed immunohistochemistry to predict response to neoadjuvant chemotherapy in the WSG-ADAPT-TN trial. J. Immunother. Cancer.

[46] Mittendorf EA (2020). Neoadjuvant atezolizumab in combination with sequential nab-paclitaxel and anthracycline-based chemotherapy versus placebo and chemotherapy in patients with early-stage triple-negative breast cancer (IMpassion031): a randomised, double-blind, phase 3 trial. Lancet.

[47] Schmid P (2020). Pembrolizumab for early triple-negative breast cancer. N. Engl. J. Med..

[48] Nanda R (2020). Effect of pembrolizumab plus neoadjuvant chemotherapy on pathologic complete response in women with early-stage breast cancer: an analysis of the ongoing phase 2 adaptively randomized I-SPY2 trial. JAMA Oncol..

[49] den Brok, W. D. et al. Homologous recombination deficiency in breast cancer: a clinical review. *JCO Precis. Oncol*. 10.1200/PO.16.00031 (2017). 10.1200/PO.16.00031 35172494

[50] Pellegrino B (2020). Homologous recombination repair deficiency and the immune response in breast cancer: a literature review. Transl. Oncol..

[51] Szekely, B. et al. Immunological differences between primary and metastatic breast cancer. *Ann. Oncol*. 10.1093/annonc/mdy399 (2018). 10.1093/annonc/mdy399 30203045

[52] Alba-Bernal A (2020). Challenges and achievements of liquid biopsy technologies employed in early breast cancer. EBioMedicine.

[53] Parker JS (2009). Supervised risk predictor of breast cancer based on intrinsic subtypes. J. Clin. Oncol..

[54] Wallden B (2015). Development and verification of the PAM50-based Prosigna breast cancer gene signature assay. BMC Med. Genomics.

[55] Brauer HA, Mashadi-Hossein A, Buckingham W, Danaher P, Ferree S (2018). Gene expression signature development to decode breast cancer heterogeneity. J. Clin. Oncol.

[56] Benjamini, Y. & Yekutieli, D. The control of the false discovery rate in multiple testing under dependency. *Ann. Stat*. 10.1214/aos/1013699998 (2001).

