# Supplementary material for: Gene signatures in patients with early breast cancer and relapse despite pathologic complete response
Source: NPJ Breast Cancer. 2022 Mar 29;8:42. doi: 10.1038/s41523-022-00403-3 (PMC8964729; doi:10.1038/s41523-022-00403-3)
Supplement: Supplementary file 1 — Supplementary Tables [file 41523_2022_403_MOESM1_ESM.pdf]

Table S1. Interindividual comparison of primary tumor gene expression between patients with relapse despite pCR and controls with pCR and no relapse, for the overall cohort and the distant relapse subgroup.

Gene/signature	**LogFC[a] (95% CI), *P* value, FDR-adjusted[b] *P* value**	
	**Any relapse**<br>patients, n = 14<br>controls, n = 40	**Distant relapse**<br>patients, n = 8<br>controls, n = 23
APM	-0.350 (-1.092, 0.392)<br>*P* = 0.348<br>Adj. *P* = 1.000	-0.822 (-1.797, 0.152)<br>*P* = 0.092<br>Adj. *P* = 1.000
Apoptosis	-0.035 (-0.225, 0.154)<br>*P* = 0.710<br>Adj. *P* = 1.000	0.177 (-0.063, 0.358)<br>*P* = 0.163<br>Adj. *P* = 1.000
AR	-0.215 (-1.502, 1.070)<br>*P* = 0.738<br>Adj. *P* = 1.000	-0.245 (-1.982, 1.491)<br>*P* = 0.775<br>Adj. *P* = 1.000
B7-H3	-0.013 (-0.496, 0.469)<br>*P* = 0.956<br>Adj. *P* = 1.000	0.426 (-0.100, 0.951)<br>*P* = 0.108<br>Adj. *P* = 1.000
BC p53	0.204 (-0.264, 0.673)<br>*P* = 0.384<br>Adj. *P* = 1.000	0.151 (-0.387, 0.688)<br>*P* = 0.572<br>Adj. *P* = 1.000
BC Proliferation	0.307 (-0.098, 0.713)<br>*P* = 0.134<br>Adj. *P* = 1.000	0.364 (-0.074, 0.801)<br>*P* = 0.101<br>Adj. *P* = 1.000
BRCAness	0.417 (-0.154, 0.988)<br>*P* = 0.149<br>Adj. *P* = 1.000	0.517 (-0.196, 1.231)<br>*P* = 0.149<br>Adj. *P* = 1.000
CD8 T-Cells	0.099 (-0.648, 0.847)<br>*P* = 0.791<br>Adj. *P* = 1.000	-0.217 (-1.040, 0.606)<br>*P* = 0.594<br>Adj. *P* = 1.000
CDK4 Expression	-0.047 (-0.325, 0.231)<br>*P* = 0.737<br>Adj. *P* = 1.000	0.014 (-0.404, 0.431)<br>*P* = 0.948<br>Adj. *P* = 1.000
CDK6 Expression	0.013 (-0.589, 0.615)<br>*P* = 0.966<br>Adj. *P* = 1.000	0.211 (-0.461, 0.883)<br>*P* = 0.526<br>Adj. *P* = 1.000
Cell Adhesion	-0.074 (-0.918, 0.771)<br>*P* = 0.862<br>Adj. *P* = 1.000	-0.173 (-0.892, 0.546)<br>*P* = 0.627<br>Adj. *P* = 1.000

Claudin-Low	0.253 (-0.651, 1.157)<br>*P* = 0.578<br>Adj. *P* = 1.000	0.178 (-0.235, 0.590)<br>*P* = 0.386<br>Adj. *P* = 1.000
Cytotoxic Cells	-0.231 (-0.979, 0.516)<br>*P* = 0.538<br>Adj. *P* = 1.000	-0.571 (-1.369, 0.225)<br>*P* = 0.153<br>Adj. *P* = 1.000
Cytotoxicity	-0.257 (-1.011, 0.496)<br>*P* = 0.496<br>Adj. *P* = 1.000	-0.565 (-1.364, 0.235)<br>*P* = 0.160<br>Adj. *P* = 1.000
Differentiation	-0.703 (-1.445, 0.040)<br>*P* = 0.063<br>Adj. *P* = 1.000	-0.453 (-1.362, 0.456)<br>*P* = 0.317<br>Adj. *P* = 1.000
Endothelial Cells	0.335 (-0.114, 0.783)<br>*P* = 0.141<br>Adj. *P* = 1.000	0.227 (-0.251, 0.705)<br>*P* = 0.340<br>Adj. *P* = 1.000
ER Signaling	-0.116 (-0.779, 0.547)<br>*P* = 0.727<br>Adj. *P* = 1.000	-0.061 (-0.845, 0.723)<br>*P* = 0.875<br>Adj. *P* = 1.000
ERBB2	0.116 (-1.481, 1.714)<br>*P* = 0.884<br>Adj. *P* = 1.000	0.318 (-1.847, 2.484)<br>*P* = 0.766<br>Adj. *P* = 1.000
ESR1	-0.274 (-1.614, 1.067)<br>*P* = 0.684<br>Adj. *P* = 1.000	-0.555 (-2.057, 0.948)<br>*P* = 0.457<br>Adj. *P* = 1.000
FOXA1	-0.719 (-2.594, 1.157)<br>*P* = 0.446<br>Adj. *P* = 1.000	-0.107 (-2.446, 2.232)<br>*P* = 0.926<br>Adj. *P* = 1.000
**HRD**	0.397 (-0.098, 0.891)<br>*P* = 0.114<br>Adj. *P* = 1.000	**0.649 (0.083, 1.214)**<br>***P* = 0.026**<br>**Adj. *P* = 1.000**
Hypoxia	0.153 (-0.250, 0.556)<br>*P* = 0.451<br>Adj. *P* = 1.000	0.478 (-0.032, 0.987)<br>*P* = 0.065<br>Adj. *P* = 1.000
IDO1	-0.552 (-1.780, 0.676)<br>*P* = 0.371<br>Adj. *P* = 1.000	-1.360 (-2.846, 0.126)<br>*P* = 0.071<br>Adj. *P* = 1.000
IFNγ	-0.279 (-1.035, 0.477)<br>*P* = 0.462<br>Adj. *P* = 1.000	-0.759 (-1.534, 0.172)<br>*P* = 0.055<br>Adj. *P* = 1.000
Inflammatory Chemokines	-0.406 (-1.056, 0.244)<br>*P* = 0.216<br>Adj. *P* = 1.000	-0.259 (-1.120, 0.602)<br>*P* = 0.543<br>Adj. *P* = 1.000

Macrophages	-0.097 (-0.471, 0.277)<br>*P* = 0.607<br>Adj. *P* = 1.000	-0.132 (-0.472, 0.207)<br>*P* = 0.432<br>Adj. *P* = 1.000
Mammary Stemness	0.445 (-0.505, 1.394)<br>*P* = 0.352<br>Adj. *P* = 1.000	0.438 (-0.392, 1.268)<br>*P* = 0.289<br>Adj. *P* = 1.000
Mast Cells	-0.201 (-1.178, 0.776)<br>*P* = 0.681<br>Adj. *P* = 1.000	0.181 (-0.912, 1.274)<br>*P* = 0.738<br>Adj. *P* = 1.000
**MHC2**	**-0.819 (-1.564, -0.073)**<br>***P* = 0.032**<br>**Adj. *P* = 1.000**	**-1.151 (-2.073, -0.229)**<br>***P* = 0.014**<br>**Adj. *P* = 1.000**
PD-1	0.116 (-0.545, 0.777)<br>*P* = 0.727<br>Adj. *P* = 1.000	-0.132 (-0.860, 0.596)<br>*P* = 0.714<br>Adj. *P* = 1.000
PD-L1	-0.182 (-0.782, 0.418)<br>*P* = 0.545<br>Adj. *P* = 1.000	-0.430 (-1.131, 0.271)<br>*P* = 0.219<br>Adj. *P* = 1.000
PD-L2	-0.224 (-0.728, 0.281)<br>*P* = 0.378<br>Adj. *P* = 1.000	-0.289 (-0.789, 0.212)<br>*P* = 0.248<br>Adj. *P* = 1.000
PR	-0.075 (-1.242, 1.091)<br>*P* = 0.898<br>Adj. *P* = 1.000	0.093 (-1.364, 1.550)<br>*P* = 0.897<br>Adj. *P* = 1.000
PTEN	-0.171 (-0.625, 0.282)<br>*P* = 0.452<br>Adj. *P* = 1.000	-0.195 (-0.777, 0.387)<br>*P* = 0.500<br>Adj. *P* = 1.000
Rb1	-0.208 (-0.526, 0.109)<br>*P* = 0.194<br>Adj. *P* = 1.000	-0.188 (-0.565, 0.189)<br>*P* = 0.318<br>Adj. *P* = 1.000
ROR	-2.675 (-12.68, 7.330)<br>*P* = 0.594<br>Adj. *P* = 1.000	-1.587 (-12.82, 9.648)<br>*P* = 0.775<br>Adj. *P* = 1.000
SOX2	-0.542 (-1.582, 0.498)<br>*P* = 0.300<br>Adj. *P* = 1.000	-0.371 (-2.056, 1.314)<br>*P* = 0.656<br>Adj. *P* = 1.000
Stroma	0.182 (-0.500, 0.864)<br>*P* = 0.595<br>Adj. *P* = 1.000	0.309 (-0.320, 0.937)<br>*P* = 0.325<br>Adj. *P* = 1.000
TGFß	0.118 (-0.263, 0.500)<br>*P* = 0.537<br>Adj. *P* = 1.000	0.159 (-0.205, 0.522)<br>*P* = 0.380<br>Adj. *P* = 1.000

TIGIT	-0.023 (-0.742, 0.696)<br>*P* = 0.949<br>Adj. *P* = 1.000	-0.632 (-1.539, 0.275)<br>*P* = 0.165<br>Adj. *P* = 1.000
TIS	-0.242 (-0.920, 0.435)<br>*P* = 0.477<br>Adj. *P* = 1.000	-0.729 (-1.478, 0.020)<br>*P* = 0.056<br>Adj. *P* = 1.000
Treg	0.051 (-0.617, 0.718)<br>*P* = 0.88<br>Adj. *P* = 1.000	-0.312 (-1.013, 0.390)<br>*P* = 0.371<br>Adj. *P* = 1.000

[a]Negative values for logFC indicate lower expression in patients with relapse versus controls.

[b]FDR-adjusted P values corrected for multiplicity according to the method of Benjamini and Yekutieli (2001).

Table S2. Interindividual comparison of primary tumor gene expression between patients with relapse despite pCR and controls with pCR and no relapse, according to patient subgroup.

	**LogFC[a] (95% CI), *P* value, FDR-adjusted[b] *P* value**	
**Gene/signature**	**HER2 positive**<br>patients, n = 5<br>controls, n = 15	**triple-negative**<br>patients, n = 5<br>controls, n = 13
APM	-0.230 (-1.396, 0.937)<br>*P* = 0.684<br>Adj. *P* = 1.000	-0.944 (-2.388, 0.501)<br>*P* = 0.185<br>Adj. *P* = 1.000
Apoptosis	0.190 (-0.103, 0.482)<br>*P* = 0.190<br>Adj. *P* = 1.000	0.003 (-0.430, 0.436)<br>*P* = 0.988<br>Adj. *P* = 1.000
AR	-0.563 (-2.226, 1.100)<br>*P* = 0.486<br>Adj. *P* = 1.000	0.300 (-1.628, 2.228)<br>*P* = 0.746<br>Adj. *P* = 1.000
B7-H3	-0.076 (-0.849, 0.697)<br>*P* = 0.839<br>Adj. *P* = 1.000	0.713 (-0.137, 1.562)<br>*P* = 0.095<br>Adj. *P* = 1.000
p53	0.366 (-0.120, 0.851)<br>*P* = 0.131<br>Adj. *P* = 1.000	-0.030 (-0.625, 0.566)<br>*P* = 0.918<br>Adj. *P* = 1.000
**Proliferation**	**0.721 (0.113, 1.328)**<br>***P* = 0.023**<br>**Adj. *P* = 1.000**	0.148 (-0.457, 0.753)<br>*P* = 0.612<br>Adj. *P* = 1.000
BRCAness	0.226 (-0.284, 0.736)<br>*P* = 0.366<br>Adj. *P* = 1.000	0.674 (-0.057, 1.405)<br>*P* = 0.068<br>Adj. *P* = 1.000
CD8 T-cells	-0.064 (-1.307, 1.178)<br>*P* = 0.915<br>Adj. *P* = 1.000	0.007 (-1.043, 1.056)<br>*P* = 0.989<br>Adj. *P* = 1.000
CDK4 expression	0.162 (-0.424, 0.748)<br>*P* = 0.569<br>Adj. *P* = 1.000	0.122 (-0.243, 0.488)<br>*P* = 0.488<br>Adj. *P* = 1.000
CDK6 expression	-0.034 (-0.644, 0.577)<br>*P* = 0.910<br>Adj. *P* = 1.000	-0.005 (-0.838, 0.827)<br>*P* = 0.989<br>Adj. *P* = 1.000

Cell adhesion	0.316 (-1.890, 2.521)<br>*P* = 0.767<br>Adj. *P* = 1.000	0.320 (-0.524, 1.163)<br>*P* = 0.434<br>Adj. *P* = 1.000
Claudin-Low	-0.604 (-3.032, 1.825)<br>*P* = 0.608<br>Adj. *P* = 1.000	0.664 (-0.182, 1.511)<br>*P* = 0.116<br>Adj. *P* = 1.000
Cytotoxic cells	-0.143 (-1.283, 0.997)<br>*P* = 0.795<br>Adj. *P* = 1.000	-0.388 (-1.662, 0.886)<br>*P* = 0.528<br>Adj. *P* = 1.000
Cytotoxicity	-0.224 (-1.262, 0.815)<br>*P* = 0.656<br>Adj. *P* = 1.000	-0.304 (-1.629, 1.022)<br>*P* = 0.634<br>Adj. *P* = 1.000
Differentiation	-0.230 (-1.341, 0.880)<br>*P* = 0.668<br>Adj. *P* = 1.000	-0.976 (-2.301, 0.350)<br>*P* = 0.138<br>Adj. *P* = 1.000
**Endothelial cells**	-0.090 (-0.806, 0.626)<br>*P* = 0.796<br>Adj. *P* = 1.000	**0.827 (0.058, 1.594)**<br>***P* = 0.036**<br>**Adj. *P* = 1.000**
ER signaling	0.448 (-0.686, 1.582)<br>*P* = 0.417<br>Adj. *P* = 1.000	-0.142 (-0.806, 0.522)<br>*P* = 0.659<br>Adj. *P* = 1.000
ERBB2	0.583 (-1.160, 2.327)<br>*P* = 0.491<br>Adj. *P* = 1.000	0.380 (-0.564, 1.323)<br>*P* = 0.406<br>Adj. *P* = 1.000
ESR1	0.643 (-1.268, 2.555)<br>*P* = 0.489<br>Adj. *P* = 1.000	0.023 (-0.881, 0.926)<br>*P* = 0.959<br>Adj. *P* = 1.000
FOXA1	0.760 (-1.001, 2.529)<br>*P* = 0.379<br>Adj. *P* = 1.000	-0.338 (-2.926, 2.250)<br>*P* = 0.786<br>Adj. *P* = 1.000
HRD	0.418 (-0.041, 0.876)<br>*P* = 0.072<br>Adj. *P* = 1.000	0.654 (-0.083, 1.391<br>*P* = 0.079<br>Adj. *P* = 1.000
Hypoxia	-0.116 (-0.718, 0.485)<br>*P* = 0.689<br>Adj. *P* = 1.000	0.596 (-0.177, 1.368)<br>*P* = 0.122<br>Adj. *P* = 1.000
IDO1	-0.645 (-2.257, 0.966)<br>*P* = 0.411<br>Adj. *P* = 1.000	-1.296 (-3.655, 1.063)<br>*P* = 0.261<br>Adj. *P* = 1.000

IFNγ	-0.316 (-1.427, 0.795)<br>*P* = 0.557<br>Adj. *P* = 1.000	-0.549 (-2.208, 1.110)<br>*P* = 0.493<br>Adj. *P* = 1.000
Inflammatory chemokines	-0.166 (-0.983, 0.651)<br>*P* = 0.676<br>Adj. *P* = 1.000	-0.113 (-1.307, 1.082)<br>*P* = 0.845<br>Adj. *P* = 1.000
Macrophages	-0.175 (-0.638, 0.288)<br>*P* = 0.438<br>Adj. *P* = 1.000	-0.230 (-0.877, 0.418)<br>*P* = 0.463<br>Adj. *P* = 1.000
**Mammary stemness**	-0.630 (-2.309, 1.049)<br>*P* = 0.441<br>Adj. *P* = 1.000	**1.716 (0.136, 3.296)**<br>***P* = 0.035**<br>**Adj. *P* = 1.000**
Mast cells	-0.595 (-2.136, 0.947)<br>*P* = 0.428<br>Adj. *P* = 1.000	0.237 (-1.289, 1.763)<br>*P* = 0.746<br>Adj. *P* = 1.000
MHC2	-0.476 (-1.755, 0.803)<br>*P* = 0.444<br>Adj. *P* = 1.000	-1.368 (-2.850, 0.114)<br>*P* = 0.068<br>Adj. *P* = 1.000
PD-1	0.348 (-0.680, 1.377)<br>*P* = 0.486<br>Adj. *P* = 1.000	0.033 (-1.016, 1.082)<br>*P* = 0.948<br>Adj. *P* = 1.000
PD-L1	-0.093 (-0.884, 0.698)<br>*P* = 0.809<br>Adj. *P* = 1.000	-0.429 (-1.499, 0.641)<br>*P* = 0.408<br>Adj. *P* = 1.000
PD-L2	-0.128 (-0.737, 0.480)<br>*P* = 0.665<br>Adj. *P* = 1.000	-0.260 (-1.214, 0.694)<br>*P* = 0.572<br>Adj. *P* = 1.000
**PR**	-0.090 (-2.225, 2.045)<br>*P* = 0.931<br>Adj. *P* = 1.000	**1.027 (0.099, 1.955)**<br>***P* = 0.032**<br>**Adj. *P* = 1.000**
PTEN	-0.248 (-0.797, 0.301)<br>*P* = 0.356<br>Adj. *P* = 1.000	0.140 (-0.743, 1.022)<br>*P* = 0.743<br>Adj. *P* = 1.000
Rb1	-0.206 (-0.509, 0.097)<br>*P* = 0.170<br>Adj. *P* = 1.000	-0.210 (-0.795, 0.376)<br>*P* = 0.459<br>Adj. *P* = 1.000
ROR	12.07 (-5.600, 29.73)<br>*P* = 0.168<br>Adj. *P* = 1.000	-8.338 (-22.70, 6.029)<br>*P* = 0.236<br>Adj. *P* = 1.000

SOX2	-1.304 (-3.324, 0.715)<br>*P* = 0.192<br>Adj. *P* = 1.000	0.117 (-1.964, 2.197)<br>*P* = 0.908<br>Adj. *P* = 1.000
Stroma	-0.407 (-1.294, 0.481)<br>*P* = 0.349<br>Adj. *P* = 1.000	1.154 (-0.129, 2.437)<br>*P* = 0.075<br>Adj. *P* = 1.000
TGFß	-0.063 (-0.602, 0.475)<br>*P* = 0.809<br>Adj. *P* = 1.000	0.420 (-0.166, 1.007)<br>*P* = 0.148<br>Adj. *P* = 1.000
TIGIT	-0.064 (-1.281, 1.153)<br>*P* = 0.914<br>Adj. *P* = 1.000	-0.470 (-1.943, 1.003)<br>*P* = 0.509<br>Adj. *P* = 1.000
TIS	-0.170 (-1.197, 0.857)<br>*P* = 0.732<br>Adj. *P* = 1.000	-0.691 (-2.053, 0.671)<br>*P* = 0.298<br>Adj. *P* = 1.000
Treg	0.063 (-1.107, 1.233)<br>*P* = 0.912<br>Adj. *P* = 1.000	-0.298 (-1.714, 1.118)<br>*P* = 0.661<br>Adj. *P* = 1.000

[a] Negative values for logFC indicate lower expression in patients with relapse versus controls.

[b]FDR-adjusted P values corrected for multiplicity according to the method of Benjamini and Yekutieli (2001).

Table S3. Intraindividual comparison of gene expression between matched primary tumors and post-pCR recurrences, for the overall cohort and the distant relapse subgroup.

**Gene/signature**	**LogFC[a] (95% CI), *P* value, FDR-adjusted[b] *P* value**	
	**Any relapse**<br>n = 14	**Distant relapse**<br>n = 8
APM	-0.741 (-1.641, 0.160)<br>*P* = 0.099<br>Adj. *P* = 1.000	-0.904 (-1.959, 0.146)<br>*P* = 0.082<br>Adj. *P* = 1.000
**Apoptosis**	-0.115 (-0.344, 0.114)<br>*P* = 0.298<br>Adj. *P* = 1.000	**-0.310 (-0.563, -0.057)**<br>***P* = 0.023**<br>**Adj. *P* = 1.000**
AR	1.264 (-0.238, 2.766)<br>*P* = 0.092<br>Adj. *P* = 1.000	1.539 (-0.726, 3.805)<br>*P* = 0.152<br>Adj. *P* = 1.000
B7-H3	-0.106 (-0.518, 0.307)<br>*P* = 0.605<br>Adj. *P* = 1.000	-0.205 (-0.717, 0.307)<br>*P* = 0.375<br>Adj. *P* = 1.000
BC p53	-0.256 (-0.836, 0.323)<br>*P* = 0.357<br>Adj. *P* = 1.000	-0.020 (-0.650, 0.609)<br>*P* = 0.940<br>Adj. *P* = 1.000
BC Proliferation	-0.328 (-0.882, 0.227)<br>*P* = 0.224<br>Adj. *P* = 1.000	-0.224 (-0.876, 0.428)<br>*P* = 0.443<br>Adj. *P* = 1.000
BRCAness	0.034 (-0.506, 0.573)<br>*P* = 0.899<br>Adj. *P* = 1.000	-0.121 (-0.967, 0.723)<br>*P* = 0.743<br>Adj. *P* = 1.000
**CD8 T-Cells**	-0.523 (-1.500, 0.455)<br>*P* = 0.269<br>Adj. *P* = 1.000	**-1.237 (-2.241, -0.058)**<br>***P* = 0.042**<br>**Adj. *P* = 1.000**
CDK4 Expression	0.000 (-0.247, 0.248)<br>*P* = 0.999<br>Adj. *P* = 1.000	-0.164 (-0.382, 0.054)<br>*P* = 0.119<br>Adj. *P* = 1.000
CDK6 Expression	0.144 (-0.126, 0.413)<br>*P* = 0.272<br>Adj. *P* = 1.000	-0.053 (-0.408, 0.301)<br>*P* = 0.732<br>Adj. *P* = 1.000
Cell Adhesion	-0.824 (-2.437, 0.790)<br>*P* = 0.290<br>Adj. *P* = 1.000	-0.049 (-1.906, 1.806)<br>*P* = 0.951<br>Adj. *P* = 1.000

Claudin-Low	0.879 (-0.459, 2.218)<br>*P* = 0.179<br>Adj. *P* = 1.000	0.104 (-1.377, 1.585)<br>*P* = 0.873<br>Adj. *P* = 1.000
Cytotoxic Cells	-0.284 (-1.360, 0.793)<br>*P* = 0.579<br>Adj. *P* = 1.000	-0.918 (-2.387, 0.549)<br>*P* = 0.183<br>Adj. *P* = 1.000
Cytotoxicity	-0.219 (-1.303, 0.865)<br>*P* = 0.670<br>Adj. *P* = 1.000	-0.845 (-2.336, 0.645)<br>*P* = 0.222<br>Adj. *P* = 1.000
Differentiation	0.145 (-0.501, 0.792)<br>*P* = 0.636<br>Adj. *P* = 1.000	0.373 (-0.748, 1.496)<br>*P* = 0.457<br>Adj. *P* = 1.000
Endothelial Cells	0.316 (-0.294, 0.926)<br>*P* = 0.284<br>Adj. *P* = 1.000	0.083 (-0.849, 1.016)<br>*P* = 0.839<br>Adj. *P* = 1.000
**ER Signaling**	**-0.595 (-0.984, -0.206)**<br>***P* = 0.006**<br>**Adj. *P* = 1.000**	**-0.487 (-0.932, -0.042)**<br>***P* = 0.036**<br>**Adj. *P* = 1.000**
ERBB2	-0.222 (-0.811, 0.366)<br>*P* = 0.429<br>Adj. *P* = 1.000	0.144 (-0.822, 1.112)<br>*P* = 0.733<br>Adj. *P* = 1.000
ESR1	-0.237 (-1.575, 1.102)<br>*P* = 0.709<br>Adj. *P* = 1.000	0.108 (-1.826, 2.043)<br>*P* = 0.898<br>Adj. *P* = 1.000
FOXA1	0.265 (-0.867, 1.396)<br>*P* = 0.622<br>Adj. *P* = 1.000	0.469 (-0.945, 1.885)<br>*P* = 0.458<br>Adj. *P* = 1.000
HRD	-0.116 (-0.519, 0.285)<br>*P* = 0.542<br>Adj. *P* = 1.000	-0.160 (-0.548, 0.228)<br>*P* = 0.362<br>Adj. *P* = 1.000
Hypoxia	0.244 (-0.135, 0.624)<br>*P* = 0.187<br>Adj. *P* = 1.000	0.014 (-0.610, 0.639)<br>*P* = 0.956<br>Adj. *P* = 1.000
IDO1	-1.166 (-2.498, 0.166)<br>*P* = 0.081<br>Adj. *P* = 1.000	-1.927 (-3.987, 0.133)<br>*P* = 0.063<br>Adj. *P* = 1.000
**IFNγ**	-0.695 (-1.700, 0.311)<br>*P* = 0.160<br>Adj. *P* = 1.000	**-1.114 (-2.208, -0.020)**<br>***P* = 0.047**<br>**Adj. *P* = 1.000**
Inflammatory Chemokines	0.348 (-0.252, 0.949)<br>*P* = 0.233<br>Adj. *P* = 1.000	-0.061 (-1.046, 0.922)<br>*P* = 0.886<br>Adj. *P* = 1.000

Macrophages	0.287 (-0.291, 0.865)<br>*P* = 0.304<br>Adj. *P* = 1.000	0.198 (-0.508, 0.905)<br>*P* = 0.528<br>Adj. *P* = 1.000
Mammary Stemness	-0.170 (-1.424, 1.085)<br>*P* = 0.775<br>Adj. *P* = 1.000	-1.104 (-2.406, 0.197)<br>*P* = 0.085<br>Adj. *P* = 1.000
Mast Cells	-0.274 (-1.790, 1.242)<br>*P* = 0.703<br>Adj. *P* = 1.000	-1.323 (-2.801, 0.154)<br>*P* = 0.072<br>Adj. *P* = 1.000
MHC2	-0.004 (-0.537, 0.529)<br>*P* = 0.988<br>Adj. *P* = 1.000	-0.385 (-0.969, 0.198)<br>*P* = 0.163<br>Adj. *P* = 1.000
PD-1	-0.310 (-1.237, 0.618)<br>*P* = 0.484<br>Adj. *P* = 1.000	-1.102 (-2.397, 0.192)<br>*P* = 0.084<br>Adj. *P* = 1.000
PD-L1	-0.250 (-1.069, 0.561)<br>*P* = 0.518<br>Adj. *P* = 1.000	-0.819 (-1.953, 0.314)<br>*P* = 0.131<br>Adj. *P* = 1.000
PD-L2	-0.125 (-1.012, 0.763)<br>*P* = 0.766<br>Adj. *P* = 1.000	-0.869 (-1.821, 0.826)<br>*P* = 0.068<br>Adj. *P* = 1.000
PR	-0.409 (-1.522, 0.705)<br>*P* = 0.442<br>Adj. *P* = 1.000	-1.061 (-2.503, 0.380)<br>*P* = 0.125<br>Adj. *P* = 1.000
PTEN	-0.013 (-0.447, 0.420)<br>*P* = 0.950<br>Adj. *P* = 1.000	0.026 (-0.647, 0.700)<br>*P* = 0.928<br>Adj. *P* = 1.000
Rb1	0.211 (-0.106, 0.527)<br>*P* = 0.175<br>Adj. *P* = 1.000	0.058 (-0.228, 0.345)<br>*P* = 0.646<br>Adj. *P* = 1.000
ROR	-3.000 (-14.13, 8.134)<br>*P* = 0.570<br>Adj. *P* = 1.000	1.875 (-11.15, 14.90)<br>*P* = 0.744<br>Adj. *P* = 1.000
SOX2	0.711 (-0.155, 1.577)<br>*P* = 0.100<br>Adj. *P* = 1.000	0.449 (-0.971, 1.869)<br>*P* = 0.479<br>Adj. *P* = 1.000
**Stroma**	-0.241 (-1.065, 0.583)<br>*P* = 0.539<br>Adj. *P* = 1.000	**-0.877 (-1.755, 0.0004)**<br>***P* = 0.050**<br>**Adj. *P* = 1.000**
TGFß	-0.441 (-0.973, 0.091)<br>*P* = 0.097<br>Adj. *P* = 1.000	-0.493 (-1.077, 0.907)<br>*P* = 0.086<br>Adj. *P* = 1.000

**TIGIT**	-1.037 (-2.129, -0.054)<br>*P* = 0.061<br>Adj. *P* = 1.000	**-1.860 (-3.375, -3.346)**<br>***P* = 0.023**<br>**Adj. *P* = 1.000**
TIS	-0.653 (-1.599, 0.293)<br>*P* = 0.160<br>Adj. *P* = 1.000	-1.150 (-2.373, 0.073)<br>*P* = 0.062<br>Adj. *P* = 1.000
**Treg**	-0.696 (-1.670, 0.279)<br>*P* = 0.147<br>Adj. *P* = 1.000	**-1.349 (-2.488, -0.210)**<br>***P* = 0.026**<br>**Adj. *P* = 1.000**

[a]Negative values for logFC indicate lower expression in recurrent versus primary tumors.

[b]FDR-adjusted P values corrected for multiplicity according to the method of Benjamini and Yekutieli (2001).

Table S4. Intraindividual comparison of gene expression between matched primary tumors and post-pCR recurrences, according to patient subgroup.

Gene/signature	LogFC[a] (95% CI), *P* value, FDR-adjusted[b] *P* value	
	**HER2 positive** n = 5	**triple-negative** n = 5
APM	-0.628 (-2.885, 1.628)<br>*P* = 0.483<br>Adj. *P* = 1.000	-0.985 (-2.197, 0.226)<br>*P* = 0.087<br>Adj. *P* = 1.000
Apoptosis	-0.143 (-0.537, 0.250)<br>*P* = 0.369<br>Adj. *P* = 1.000	-0.307 (-0.873, 0.258)<br>*P* = 0.206<br>Adj. *P* = 1.000
AR	0.640 (-2.992, 4.273)<br>*P* = 0.650<br>Adj. *P* = 1.000	2.675 (-0.903, 6.255)<br>*P* = 0.107<br>Adj. *P* = 1.000
B7-H3	-0.070 (-1.270, 1.128)<br>*P* = 0.878<br>Adj. *P* = 1.000	-0.369 (-0.995, 0.256)<br>*P* = 0.177<br>Adj. *P* = 1.000
p53	0.198 (-0.623, 1.021)<br>*P* = 0.539<br>Adj. *P* = 1.000	-0.706 (-2.154, 0.741)<br>*P* = 0.247<br>Adj. *P* = 1.000
Proliferation	0.028 (-0.882, 0.939)<br>*P* = 0.935<br>Adj. *P* = 1.000	-0.896 (-2.196, 0.404)<br>*P* = 0.128<br>Adj. *P* = 1.000
BRCAness	0.721 (-0.022, 1.464)<br>*P* = 0.054<br>Adj. *P* = 1.000	-0.542 (-1.464, 0.379)<br>*P* = 0.178<br>Adj. *P* = 1.000
CD8 T-Cells	-0.899 (-2.574, 0.774)<br>*P* = 0.210<br>Adj. *P* = 1.000	-1.109 (-3.047, 0.841)<br>*P* = 0.190<br>Adj. *P* = 1.000
CDK4 expression	-0.064 (-0.641, 0.513)<br>*P* = 0.773<br>Adj. *P* = 1.000	-0.217 (-0.664, 0.229)<br>*P* = 0.248<br>Adj. *P* = 1.000
CDK6 expression	0.014 (-0.594, 0.623)<br>*P* = 0.950<br>Adj. *P* = 1.000	0.326 (-0.010, 0.663)<br>*P* = 0.055<br>Adj. *P* = 1.000
Cell adhesion	0.551 (-0.976, 2.080)<br>*P* = 0.373<br>Adj. *P* = 1.000	0.627 (-1.273, 2.528)<br>*P* = 0.411<br>Adj. *P* = 1.000

Claudin-Low	0.175 (-1.461, 1.811)<br>*P* = 0.781<br>Adj. *P* = 1.000	-0.352 (-0.841, 0.136)<br>*P* = 0.116<br>Adj. *P* = 1.000
Cytotoxic cells	-0.818 (2.209, 1.272)<br>*P* = 0.338<br>Adj. *P* = 1.000	-0.611 (-2.845, 1.621)<br>*P* = 0.489<br>Adj. *P* = 1.000
Cytotoxicity	-0.926 (-2.934, X.XXX)<br>*P* = 0.269<br>Adj. *P* = 1.000	-0.652 (-2.916, 1.610)<br>*P* = 0.468<br>Adj. *P* = 1.000
Differentiation	0.414 (-1.588, 2.417)<br>*P* = 0.597<br>Adj. *P* = 1.000	0.267 (-0.793, 1.328)<br>*P* = 0.522<br>Adj. *P* = 1.000
Endothelial cells	-0.201 (-1.674, 1.272)<br>*P* = 0.724<br>Adj. *P* = 1.000	0.424 (-0.452, 1.301)<br>*P* = 0.250<br>Adj. *P* = 1.000
**ER signaling**	-0.922 (-1.992, 0.146)<br>*P* = 0.075<br>Adj. *P* = 1.000	**-0.405 (-1.205, 0.394)**<br>***P* = 0.232**<br>**Adj. *P* = 1.000**
ERBB2	-0.076 (-1.276, 1.122)<br>*P* = 0.867<br>Adj. *P* = 1.000	0.417 (-0.272, 1.107)<br>*P* = 0.169<br>Adj. *P* = 1.000
**ESR1**	**-1.804 (-3.567, -0.040)**<br>***P* = 0.047**<br>**Adj. *P* = 1.000**	1.596 (-1.237, 4.431)<br>*P* = 0.193<br>Adj. *P* = 1.000
FOXA1	-0.533 (-1.578, 0.511)<br>*P* = 0.229<br>Adj. *P* = 1.000	1.907 (0.030, 3.845)<br>*P* = 0.052<br>Adj. *P* = 1.000
HRD	0.281 (-0.280, 0.842)<br>*P* = 0.237<br>Adj. *P* = 1.000	-0.646 (-1.459, 0.165)<br>*P* = 0.092<br>Adj. *P* = 1.000
Hypoxia	0.584 (-0.060, 1.229)<br>*P* = 0.066<br>Adj. *P* = 1.000	-0.177 (-1.105, 0.751)<br>*P* = 0.624<br>Adj. *P* = 1.000
IDO1	-1.285 (-5.514, 2.942)<br>*P* = 0.446<br>Adj. *P* = 1.000	-1.398 (-3.478, 0.680)<br>*P* = 0.135<br>Adj. *P* = 1.000
IFNγ	-0.517 (-2.890, 1.855)<br>*P* = 0.577<br>Adj. *P* = 1.000	-1.097 (-2.698, 0.502)<br>*P* = 0.130<br>Adj. *P* = 1.000

Inflammatory chemokines	-0.115 (-1.544, 1.313)<br>*P* = 0.833<br>Adj. *P* = 1.000	-0.002 (-0.962, 0.958)<br>*P* = 0.995<br>Adj. *P* = 1.000
Macrophages	0.293 (-0.787, 1.373)<br>*P* = 0.494<br>Adj. *P* = 1.000	0.097 (-1.047, 1.242)<br>*P* = 0.824<br>Adj. *P* = 1.000
Mammary stemness	-0.415 (-3.818, 2.986)<br>*P* = 0.751<br>Adj. *P* = 1.000	-0.805 (-2.183, 0.572)<br>*P* = 0.180<br>Adj. *P* = 1.000
Mast cells	-0.442 (-4.556, 3.671)<br>*P* = 0.780<br>Adj. *P* = 1.000	-0.188 (-2.569, 2.191)<br>*P* = 0.836<br>Adj. *P* = 1.000
MHC2	-0.048 (-1.551, 1.453)<br>*P* = 0.933<br>Adj. *P* = 1.000	-0.132 (-0.993, 0.728)<br>*P* = 0.691<br>Adj. *P* = 1.000
PD-1	-0.681 (-2.079, 0.716)<br>*P* = 0.247<br>Adj. *P* = 1.000	-0.988 (-3.354, 1.377)<br>*P* = 0.310<br>Adj. *P* = 1.000
PD-L1	-0.532 (-2.539, 1.475)<br>*P* = 0.503<br>Adj. *P* = 1.000	-0.490 (-2.324, 1.344)<br>*P* = 0.499<br>Adj. *P* = 1.000
PD-L2	-0.420 (-2.782, 1.941)<br>*P* = 0.647<br>Adj. *P* = 1.000	-0.395 (-1.628, 0.836)<br>*P* = 0.423<br>Adj. *P* = 1.000
PR	-1.065 (-3.845, 1.714)<br>*P* = 0.347<br>Adj. *P* = 1.000	0.088 (-0.888, 1.064)<br>*P* = 0.814<br>Adj. *P* = 1.000
**PTEN**	**-0.442 (-0.864, -0.019)**<br>***P* = 0.044**<br>**Adj. *P* = 1.000**	0.269 (-0.829, 1.368)<br>*P* = 0.533<br>Adj. *P* = 1.000
Rb1	0.157 (-0.314, 0.629)<br>*P* = 0.406<br>Adj. *P* = 1.000	0.147 (-0.360, 0.655)<br>*P* = 0.466<br>Adj. *P* = 1.000
ROR	-5.400 (-37.59, 26.79)<br>*P* = 0.666<br>Adj. *P* = 1.000	-8.200 (-26.35, 9.951)<br>*P* = 0.278<br>Adj. *P* = 1.000
SOX2	1.651 (-0.026, 3.329)<br>*P* = 0.052<br>Adj. *P* = 1.000	-0.205 (-1.883, 1.471)<br>*P* = 0.750<br>Adj. *P* = 1.000

**Stroma**	-0.111 (-2.246, 2.023)<br>*P* = 0.892<br>Adj. *P* = 1.000	**-1.040 (-1.755, -0.325)**<br>***P* = 0.016**<br>**Adj. *P* = 1.000**
**TGFß**	**-0.503 (-0.932, -0.074)**<br>***P* = 0.031**<br>**Adj. *P* = 1.000**	**-0.673 (-1.008, -0.339)**<br>***P* = 0.005**<br>**Adj. *P* = 0.909**
TIGIT	-1.246 (-3.712, 1.219)<br>*P* = 0.233<br>Adj. *P* = 1.000	-1.628 (-4.152, 0.895)<br>*P* = 0.148<br>Adj. *P* = 1.000
TIS	-0.810 (-2.968, 1.347)<br>*P* = 0.356<br>Adj. *P* = 1.000	-0.970 (-2.815, 0.873)<br>*P* = 0.218<br>Adj. *P* = 1.000
Treg	-0.809 (-2.375, 1.304)<br>*P* = 0.464<br>Adj. *P* = 1.000	-1.141 (-3.409, 1.126)<br>*P* = 0.235<br>Adj. *P* = 1.000

[a]Negative values for logFC indicate lower expression in recurrent versus primary tumors.

[b]FDR-adjusted P values corrected for multiplicity according to the method of Benjamini and Yekutieli (2001).
